# Ring distributions leading to species formation: a global topographic analysis of geographic barriers associated with ring species

**DOI:** 10.1186/1741-7007-10-20

**Published:** 2012-03-12

**Authors:** William B Monahan, Ricardo J Pereira, David B Wake

**Affiliations:** 1 Museum of Vertebrate Zoology, 3101 Valley Life Sciences Building, University of California, Berkeley, CA 94720, USA; 2 National Park Service, Inventory and Monitoring Division, 1201 Oakridge Drive, Suite 150, Fort Collins, CO 80525, USA; 3 CIBIO, Centro de Investigação em Biodiversidade e Recursos Genéticos, Universidade do Porto, Campus Agrário de Vairão, 4485-661 Vairão, Portugal; 4 Department of Integrative Biology, University of California, Berkeley, CA 94720, USA

## Abstract

**Background:**

In the mid 20^th ^century, Ernst Mayr and Theodosius Dobzhansky championed the significance of circular overlaps or ring species as the perfect demonstration of speciation, yet in the over 50 years since, only a handful of such taxa are known. We developed a topographic model to evaluate whether the geographic barriers that favor processes leading to ring species are common or rare, and to predict where other candidate ring barriers might be found.

**Results:**

Of the 952,147 geographic barriers identified on the planet, only about 1% are topographically similar to barriers associated with known ring taxa, with most of the likely candidates occurring in under-studied parts of the world (for example, marine environments, tropical latitudes). Predicted barriers separate into two distinct categories: (i) single cohesive barriers (< 50,000 km^2^), associated with taxa that differentiate at smaller spatial scales (salamander: *Ensatina eschscholtzii*; tree: *Acacia karroo*); and (ii) composite barriers - formed by groups of barriers (each 184,000 to 1.7 million km^2^) in close geographic proximity (totaling 1.9 to 2.3 million km^2^) - associated with taxa that differentiate at larger spatial scales (birds: *Phylloscopus trochiloide*s and *Larus *(sp. *argentatus *and *fuscus*)). When evaluated globally, we find a large number of cohesive barriers that are topographically similar to those associated with known ring taxa. Yet, compared to cohesive barriers, an order of magnitude fewer composite barriers are similar to those that favor ring divergence in species with higher dispersal.

**Conclusions:**

While these findings confirm that the topographic conditions that favor evolutionary processes leading to ring speciation are, in fact, rare, they also suggest that many understudied natural systems could provide valuable demonstrations of continuous divergence towards the formation of new species. Distinct advantages of the model are that it (i) requires no *a priori *information on the relative importance of features that define barriers, (ii) can be replicated using any kind of continuously distributed environmental variable, and (iii) generates spatially explicit hypotheses of geographic species formation. The methods developed here - combined with study of the geographical ecology and genetics of taxa in their environments - should enable recognition of ring species phenomena throughout the world.

## Background

Polytypic species and complexes of closely related species provide unusual opportunities to study the linkage between micro and macro evolutionary processes directly in nature because they are composed of taxa that persist at various stages of divergence, from genetically differentiated populations to ecologically divergent taxa. Of particular importance are ring species [1], or circular overlaps [2], in which populations at intermediate stages of divergence are distributed around a geographic barrier and reconnect at a terminus as reproductively isolated taxa. By preserving genetic interactions that are typical of species at the ring terminus, as well as interactions typical of populations around the ring distribution, these systems provide a natural demonstration of how micro-evolutionary processes (that is, colonization, genetic drift, gene flow, and local adaptation) result in a continuum of divergence, linking taxa that are generally recognized as species. Although prized as examples of evolutionary clarity, ring species also present a pattern of taxonomic irresolution in which, facing continuous levels of differentiation, different taxonomists recognize a varying number of species, depending on their criteria. Most previous studies of ring species have focused on the local geographical and ecological factors enabling species formation. Here, we develop a generalized model of geographic barriers and use the known examples of ring species to evaluate the number and distribution of other barriers from around the world that are topographically similar and thus may be promoting ring speciation processes in equivalent taxa.

Geographic species formation is intrinsically dependent on the spatial scale at which organisms interact with the landscape, encompassing both biological and historical factors that affect divergence (for example, age of the clade, generation time), and others that affect homogenization through gene flow (for example, degree of philopatry, rate and distance of successful migration, home range size) (see [3]). Theoretically, ring species can arise frequently when the spatial scale of a geographic barrier matches the biological and historical 'scales' that are necessary for species-level divergence [4,5]. Whether because that ratio is rarely met in nature or because of historical contingencies associated with the barrier or the organism, few polytypic taxa are in fact recognized by modern taxonomy as ring species [6] and ring diversification is considered to be the exceptional mode of geographic diversification [7]. Mayr [8] stated that "circular overlaps can obviously develop only under highly exceptional constellations of geographical factors", so that the continuous levels of population divergence result from restrictions to gene flow within a species' range imposed by a central and long-standing geographic barrier. Despite their apparent rarity, ring species were extremely influential to the Evolutionary Synthesis [2,9] and remain a cornerstone to our understanding of how geography influences species formation. These few examples seem to indicate that - even though species formation is clearly a continuous process [10] - the geographic conditions that promote ring speciation are extremely rare. Perhaps there is a taxonomic impediment, in which discovery of parts of rings and their naming as species precedes (as in the case of the history of the *Ensatina *ring prior to its recognition as a ring [11]) or, perhaps more commonly, impedes recognition of the ring. In this paper, we release ourselves from existing taxonomic classifications, and possible related artifacts, in order to consider the processes that have enabled ring-distributed taxa ('ring taxa') to diversify in a continuous sense around geographic barriers, irrespective of whether the terminal forms are above or below species-level divergence.

Long-term research programs on ring species complexes, such as the plethodontid salamander *Ensatina eschscholtzii *and the greenish warbler *Phylloscopus trochiloide*s, provide empirical insights into the processes that can drive ring species formation: (i) conditioned by a long-standing geographic barrier, an ancestor expands around the barrier to form a ring distribution, (ii) restrictions to dispersal imposed by the barrier are such that contiguous populations become increasingly more divergent, and (iii) this divergence continues to the point where - at the ring terminus - the reconnecting terminal taxa are reproductively isolated or hybridize infrequently (that is, without an opportunity for gene flow). The persistence of the central geographic barrier is fundamental for ring diversification because it restricts movement of individuals to the ring distribution, thus promoting non-adaptive divergence through the initial colonization of available habitat, genetic drift of each local population, and limiting gene flow among continuous populations around the ring. Adaptive divergence may further affect neighboring populations around the ring distribution through such processes as local adaptation of anti-predatory strategies (for example, coloration in *E. eschscholtzii*; [11]) or the development of assortative mating (for example, song and coloration in *P. trochiloide*s; [12]). While taxon-based studies have contributed to our understanding of the evolutionary processes that result in ring species, they are not easily generalized and thus cannot be used to evaluate the number and distribution of other geographic barriers around the world that may also favor continuous divergence in ring distributed taxa, so that terminal overlapping forms are near species-level divergence.

**Figure 1:**
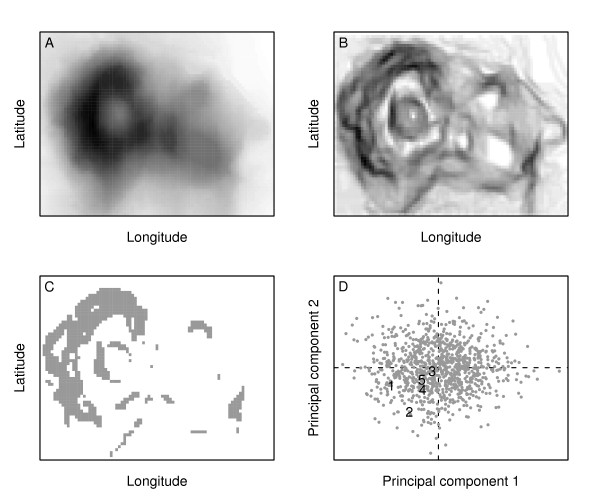
**Conceptual overview of the four-step modeling framework for quantifying geographic barriers formed by environmental gradients. A**: Our generalized model is parameterized for both marine and terrestrial environments using elevation. **B**: We then compute slope, or the spatial rate of change in elevation, which mechanistically is designed to capture environmental transitions that impose either direct or indirect barriers to species distribution. **C**: Considering all possible candidate barriers that emerge under different rates of change, we compute a series of biologically informative summary statistics that follow directly from predictions of geographical ecology and speciation and allow us to quantitatively characterize the topographic traits known to be associated with ring species formation (Table 1). **D**: Finally, we use the summary statistics for all candidate barriers in a multivariate analysis to explicitly compare among known reference barriers and identify similar candidate barriers that may be promoting similar evolutionary processes. Data for illustration purposes (A-C) provided by R Development Core Team [35].

Here, we advance a new modeling framework to address the general question of which geographic barriers provide the topographic 'canvas' necessary for the establishment of ring species (Figure 1). Rather than modeling ring distributions of species, our predictive model targets geographic barriers that, according to biologically relevant summary statistics (Table 1), are topographically similar to barriers associated with ring species, or taxa with a similar diversification process (that is, ring taxa that express continuous levels of differentiation, with terminal forms being above or below species level divergence). By removing any subjective and ill-defined considerations of what constitutes a valid biogeographic barrier for species, our model simplifies environmental complexity so that barrier similarities may be quantified and evaluated consistently across taxa and environments. While 'environment' in our model can be defined according to a number of different variables, here we parameterize it using elevation because it is a major topographic variable leading to the formation of prominent ecotones and defined ecoregions [13], which in turn are broad-scale determinants of species' distributions [14]. First, we analyze a multivariate 'barrier-space' to evaluate whether barriers associated with ring taxa share topographic features, and whether those features are in fact rare when considered relative to all other barriers on the planet. Second, we use our model to identify candidate barriers with equivalent topographic features to those associated with ring taxa, and for taxa with comparable population biologies and histories provide spatially explicit hypotheses of where ring diversification might be occurring in nature.

**Table 1:** Summary statistics used in the topographic ring model, along with a brief description of biological relevance.

Category	Summary statistic	Biological relevance
Size	1. Area 2. Perimeter	Larger barriers provide more opportunities for isolation by distance to promote non-adaptive divergence (that is, differentiation in neutral loci) around a ring distribution.
	3. Latitudinal range	Larger latitudinal ranges span more environments and thus facilitate adaptive divergence.
Position	4. Mean distance from equator	Barriers further from the equator are larger to account for latitudinal differences in range size [31].
Permeability	5. Shape (Perimeter-to-area ratio)	Compact circular-shaped barriers (compared to elongated barriers) are uniformly wider and therefore less subject to trans-barrier dispersal and gene flow.
	6. Fragmentation	More fragmented barriers (that is, barriers that split apart with changing topographic slope) offer more opportunities for trans-barrier dispersal than uniform barriers.

## Results and discussion

### Reference ring taxa do not always encircle single topographic barriers

**Figure 2:**
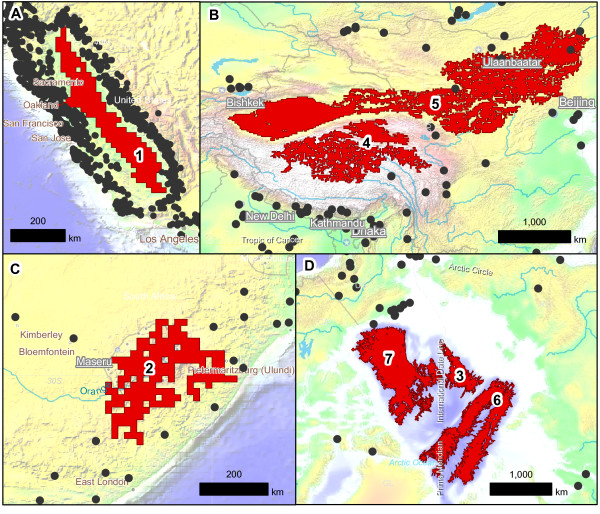
**Reference barriers and reference taxa. A**: The salamander species *Ensatina eschscholtzii*: Central Valley, California, USA. **B**: The bird species *Phylloscopus trochiloides*: Central Asia. **C**: The tree species *Acacia karroo*: Drakensberg Massif, South Africa. **D**: The bird species complex *Larus*: Arctic Ocean. Barriers are shown by the red polygons, species' distributions by the black points, and global elevations by the shaded topography. Numbers correspond to individual barriers identified in the PCA.

Using a standardized set of criteria to formally define a barrier, our topographic model identified a total of 952,147 geographically cohesive barriers on the planet. Many of these barriers aligned with ecoregions (Additional file 1), suggesting that barriers predicted by our model do indeed delineate areas that represent valid biogeographic barriers. Furthermore, when overlaying the distributions of reference ring taxa, we find that they encircle topographic barriers identified by our model (Figure 2). However, contrary to expectations based on both classical [2,8] and recent [6] reviews of ring species, at the spatial scale of our analysis not all ring taxa encircle single topographic barriers. While *Ensatina *and *Acacia *encircle single barriers ('cohesive barriers'; Figure 2A, C), the bird taxa *Larus *and *Phylloscopus *encircle two or more barriers that are in close geographic proximity ('composite barriers'; Figure 2B, D). The seven barriers associated with our focal ring taxa align with major topographic features that define important climatic and ecological transitions (numbers correspond to barriers labeled in the PCA, below) are: (1) *Ensatina*: Central Valley, California, USA; (2) *Acacia*: Drakensberg Massif, South Africa; (3) *Larus*: Makarov Basin, Arctic Ocean; (4) *Phylloscopus*: Tibetan Plateau, Central Asia; (5) *Phylloscopus*: Takla Maka and Gobi Deserts, Central Asia; (6) *Larus*: Amundsen and Nansen Basins, Arctic Ocean; (7) *Larus*: Canada Basin, Arctic Ocean. The close spatial proximity of the individual barriers associated with the bird taxa (3 to 7) relate to larger topographic features generally known as Central Asia (barriers 4 and 5) and the Arctic Ocean (barriers 3, 6 and 7). In addition to *Larus *and *Phylloscopus*, these composite barriers have also contributed to ring divergence in three other birds (Additional file 2).

Our finding that composite barriers exist and can promote ring diversification even in taxa that disperse widely is not simply an artifact of the model or the spatial resolution of the data. At different spatial resolutions of topographic slope (30 arc sec to 3 arc degrees) the model still predicted Central Asia and the Arctic Ocean as composite barriers. Furthermore, composite barriers encompass such large geographic areas (millions of km^2 ^and hundreds of different ecoregions) that it is difficult to imagine any univariate or multivariate environmental approximation of a single barrier (for example, Central Asia, which is comprised of the Takla Maka-Gobi deserts and the Tibetan Plateau - large geographic regions that differ dramatically in terms of climate and vegetation). If ring taxa that disperse widely are in fact distributed around composite barriers, then an important implication for ring speciation is that individual barriers in close spatial proximity can interact with one another to form effective barriers to species distribution that are orders of magnitude larger than any single cohesive barrier. More empirical work is required to determine the spatial characteristics of inter-barrier gaps that prevent ring taxa from maintaining genetic connectivity across composite barriers.

### Barriers associated with ring taxa share topographic features that are rarely found in nature

**Figure 3:**
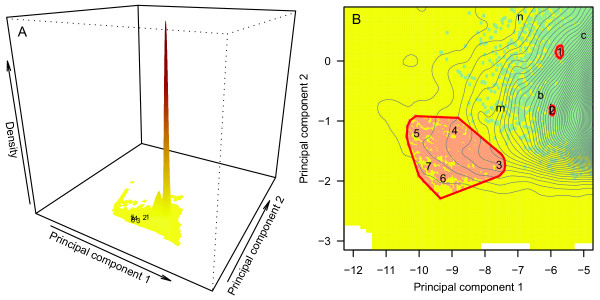
**Principal component analysis of geographic barriers identified by the topographic ring model. A**: Density of all candidate barriers on the planet, with numbers corresponding to reference barriers known to promote ring speciation processes: (1) *Ensatina*: Central Valley, California, USA; (2) *Acacia*: Drakensberg Massif, South Africa; (3) *Larus*: Makarov Basin, Arctic Ocean; (4) *Phylloscopus*: Tibetan Plateau, Central Asia; (5) *Phylloscopus*: Takla Maka and Gobi Deserts, Central Asia; (6) *Larus*: Amundsen and Nansen Basins, Arctic Ocean; (7) *Larus*: Canada Basin, Arctic Ocean. Darker red tones correspond to higher barrier density; whitespace identifies areas where barriers are undefined (that is, non-existent). **B**: Vertical zoom on numbers 1 to 7 in A, showing in red the 100 closest cohesive candidate barriers (Euclidean distance) to the *Ensatina *and *Acacia *reference barriers (1 and 2), and the 1,380 cohesive candidate barriers identified for the bird taxa *Larus *and *Phylloscopus *and used to evaluate candidate composite barriers. Green points show the distribution of barriers where permeability as measured by fragmentation was less than the maximum possible. Lowercase letters show the locations of barriers evaluated in the discussion (b = Baja, c = Costa Rica, i = Iberian Peninsula, m = Madagascar, n = New Guinea). Black contours identify (from left to right) tiers of increasing density.

All barriers identified globally by our model are shown in a multivariate topographic space (Figure 3A), computed using a principal component analysis on the six barrier summary statistics (Table 1). Principal component (PC) 1 explained 64% of the variation and loaded most heavily on size (area, perimeter, latitudinal range) and permeability (shape); PC2 explained an additional 19% of the variation and loaded most heavily on position (distance from equator) and permeability (fragmentation; Additional file 3).

Considering all geographic barriers identified globally by our model, most share topographic features that cause them to cluster in two high density peaks of the PCA (Figure 3A). Meanwhile, the seven reference barriers cluster in a very discrete, low density area of the PCA (Figure 3B). Despite known species idiosyncrasies, our model is evidently tracking barrier traits that exert effects (Table 1) across taxa, since barriers involved in ring diversification in salamander and tree taxa are near one another in multivariate space (points 1 and 2, Figure 3A), and all individual barriers that comprise composite bird barriers also cluster (points 3 to 7, Figure 3A). This result indicates that barriers associated with ring taxa share similar topographic features, and that these topographic features are relatively rare on the planet. While our small sample size prevents any formal statistical comparison, the topographic features of the barriers associated with the reference taxa are also distributed along an axis of dispersal behavior (Figure 3B). As expected, our model shows that ring taxa with higher dispersal (points 3 to 7, Figure 3B) require larger barriers than lower dispersers (points 1 and 2, Figure 3B). A better understanding of how characteristics of the barriers scale with the biology of the organism will benefit from the discovery of new ring taxa, which can fill in the biological continuum that is encompassed by the current reference ring taxa, and also expand model predictions.

Compared to the most common barriers on the planet, reference barriers are larger in size (area, perimeter, latitudinal range) and less permeable (shape). These results support initial predictions for four of our six summary statistics (Table 1). Notable exceptions include the two summary statistics on PC2: (i) position (distance from equator), where the small number of known ring species prevented us from evaluating whether larger barriers would be required at higher latitudes, and (ii) fragmentation, where we expected more fragmented barriers to allow trans-barrier gene flow and thus prevent ring diversification. In actuality, our measure of fragmentation is reflecting the real topographic complexity of barriers. While small to medium sized barriers (< 50,000 km^2^) can have lower values for fragmentation (green points in Figure 3B), larger barriers are more likely to encompass fragmenting features like valleys and ridges, so that fragmentation is maximum for all barriers above 50,000 km^2 ^(Additional file 4). For the same reason, our model recognizes larger topographic features such as the Arctic Ocean and Central Asia as clusters of individual barriers that are so large that they can no longer remain cohesive. In contrast, permeability of the barrier as measured by shape (perimeter-to-area ratio), which loaded heavily on PC1, fully matched our initial predictions, suggesting that this summary statistic might better reflect trans-barrier dispersal, or that its effect is biologically more meaningful than a finer fragmentation of the barrier. Although shape as computed by a perimeter-to-area ratio scales with size, the reference barriers were some of the most geometrically compact barriers within their respective size classes (low perimeter-to-area ratios in Additional file 4).

A minimum bounding box around the seven reference barriers (Figure 3B) encompassed approximately 1% of all candidate barriers on the planet. This statistic taken at face value suggests that geographic barriers with similar topographic characteristics to those that promote ring divergence are exceedingly rare. However, a statistic of 1% also results in about 10,000 individual cohesive barriers that are similar in terms of size, position and permeability to known ring barriers. In agreement with Mayr's [8] assertion, those topographic conditions are indeed rare when compared to all candidate barriers on the planet, but numerous when considered relative to the handful of ring species that are well-described by science [2,6,8]. This further raises the possibility that a relatively large number of under-studied barriers on the planet may be associated with taxa that are evolving under ring processes of divergence.

### Opportunities for ring divergence are more common around smaller barriers

**Figure 4:**
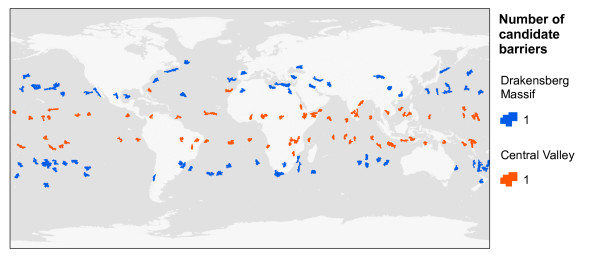
**The top 100 candidate cohesive barriers**. Candidates are topographically similar to the reference barrier for the Drakensberg Massif (South Africa), which has promoted ring diversification in the tree species *Acacia karroo*, and to the reference barrier for the Central Valley, California (USA), which has promoted ring diversification in the salamander species *Ensatina eschscholtzii*.

Candidate cohesive barriers were identified based solely on their topographic similarity to the two cohesive reference barriers associated with ring diversification in lower dispersing taxa (*Ensatina*: Central Valley, California; *Acacia*: Drakensberg Massif, South Africa). When mapped back into geographic space, these candidates are predicted throughout tropical to temperate latitudes (Figure 4). This latitudinal bias is due to the influence of position (distance from equator; Table 1) in the PCA (Additional file 3). The similarity level chosen (100 most similar barriers, Euclidean distance) retrieved spatially unique candidate cohesive barriers that were topographically equivalent to the reference barriers. Less stringent similarity levels (for example, 500 most similar barriers) would recover candidates at similar latitudes, but with a greater likelihood of spatial overlap. Because reference cohesive barriers are located in a portion of the PCA where barrier density is relatively high (Figure 3B), irrespective of one's choice of similarity threshold there are a relatively large number of individual barriers on the planet that are capable of promoting ring divergence. These candidate cohesive barriers should be evaluated in the context ring-distributed taxa with lower dispersal tendencies or, more generally, with population biologies or histories that are broadly similar to *Acacia *or *Ensatina*.

**Figure 5:**
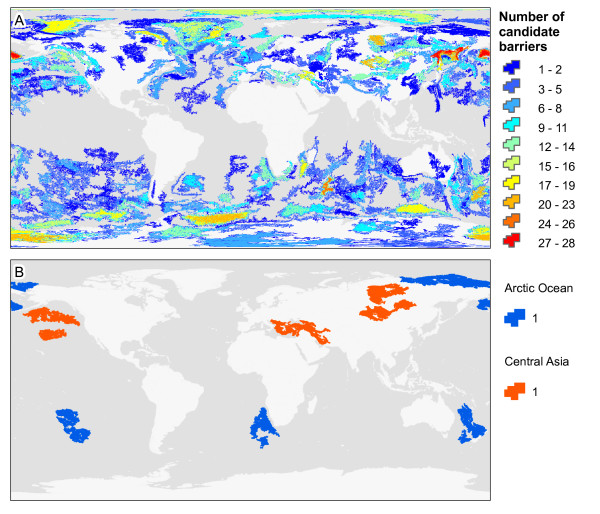
**Candidate composite barriers. A**: The top 1,380 candidate barriers that are topographically similar to the individual barriers that comprise the reference barrier for the Arctic Ocean, which has promoted ring diversification in the bird species complex *Larus*, and to the individual barriers that comprise the reference barrier for Central Asia, which has promoted which has promoted ring diversification in the bird species *Phylloscopus trochiloides*. **B**: Candidate composite barriers evaluated for groups of individual barriers showing the same number of sub-units (that is, cohesive barriers), the same inflection, and that are similar in total size and inter-barrier proximity. Seven of the 14 candidate composite barriers are shown to illustrate exemplary barriers and simplify map interpretation; the other candidates are provided in our model database (see Methods).

In contrast to cohesive barriers, candidate composite barriers were identified in two steps by choosing, (i) topographically similar individual barriers to those from reference composite barriers and (ii) combinations of individual barriers with emergent properties similar to reference composite barriers (number of barriers, total area, geographic proximity and inflection). Step 1 recovered 1,380 barriers that were topographically similar to the five barriers associated with reference bird taxa (red polygon in Figure 3B). When mapped back into geographic space, these candidates are predicted throughout sub-tropical to polar latitudes, with different degrees of spatial overlap (Figure 5A). The overlap is due to a combination of the relatively large number of candidate cohesive barriers considered, their large size (> 184,000 km^2^), and their sensitivity to fragmentation (Figure 3B). Despite the large number of candidate composite barriers formed by combinations of two to three individual barriers (respectively 951,510 and 875 million), only 14 meet the criteria of similarity to the reference composite barriers (Figure 5B). The number of candidates did not increase significantly with less stringent similarity levels (for example, threshold of 10%). Hence, at the larger spatial scales characteristic of composite barriers and higher dispersing ring taxa, very few areas on the planet present the topographic conditions associated with ring diversification. Furthermore, because higher dispersing ring taxa can maintain panmixia over such large geographic areas, few will be expected to reach species-level divergence. For example, the existence of a prolonged pelagic life stage in some marine taxa can potentially increase the homogenizing role of gene flow, so that even after circumpolar divergence the terminal taxa do not develop reproductive barriers, and merge at the terminus of the ring distribution (for example, trumpetfishes [15]).

When considered in the context of composite barriers, our model corroborates Mayr's [8] assertion that few areas of the world present the topographic conditions necessary for ring speciation. However, when considered in the context of cohesive barriers, our model also suggests that a surprisingly large number of candidate barriers exist and merit further study. One possible explanation for this discrepancy is that - while most contemporary phylogeographic studies have explored well-developed parts of the globe, like Europe and North America, the barriers most likely to be associated with ring divergence are located in under-studied regions (for example, marine environments, tropical latitudes) where new species continue to be described, their geographic ranges are still being mapped and genetic data are rare. An alternative explanation is that some of those areas have been studied but ring diversification has brought taxa to a stage when they are not clearly recognized as 'ring species'. Instead, recently diverged taxa might express continuous variation at the population level, whereas segments of older taxa might have already 'decayed' into a ring of closely related species (see [16]), thus making it unlikely that researchers would detect a near continuum of differentiation. Therefore, by providing spatially explicit phylogeographic hypotheses that can be tested with adequate genetic or phenotypic data, the topographic ring model is designed to advance field studies of species formation in ring-like patterns.

### The topographic model generates spatially explicit hypotheses that may be tested in nature

For both cohesive and composite barriers, our topographic model produces spatially explicit predictions of diversification across taxa and environments. Depending on one's chosen taxonomic criteria, whether these candidate barriers affect taxa that presently constitute valid ring species or, more generally, ring distributed taxa in which terminal forms are above or below species-level divergence, is a question that can now be addressed. Yet, the candidate barriers predicted by our model are hypothesized to result in taxa expressing continuous degrees of adaptive and non-adaptive divergence, thus allowing these processes to be investigated directly in the field. Variation in biology and history will determine the level of divergence reached by taxa that are evolving under a ring diversification process, that is, whether taxa are currently recognized as a ring of populations, a ring species or a ring of species.

Establishing whether taxa comprise a valid ring species ultimately requires extensive population-level sampling around the ring distribution to test for increasing levels of divergence between contiguous populations (for example, [12,17]), and restricted genetic interaction in secondary contacts across the ring when compared to contacts around the ring [18]. However, there are three lines of evidence with respect to the candidate barrier that may be considered prior to investing in such detailed population-level sampling: barrier topographic traits, associated environmental gradients and species' distributions. We illustrate these in combination for a barrier in Costa Rica and Panama that, while being a mountain barrier, is topographically similar in size, shape and permeability to the Central Valley of California, which has promoted ring diversification in the salamander *Ensatina eschscholtzii *[11,19].

**Figure 6:**
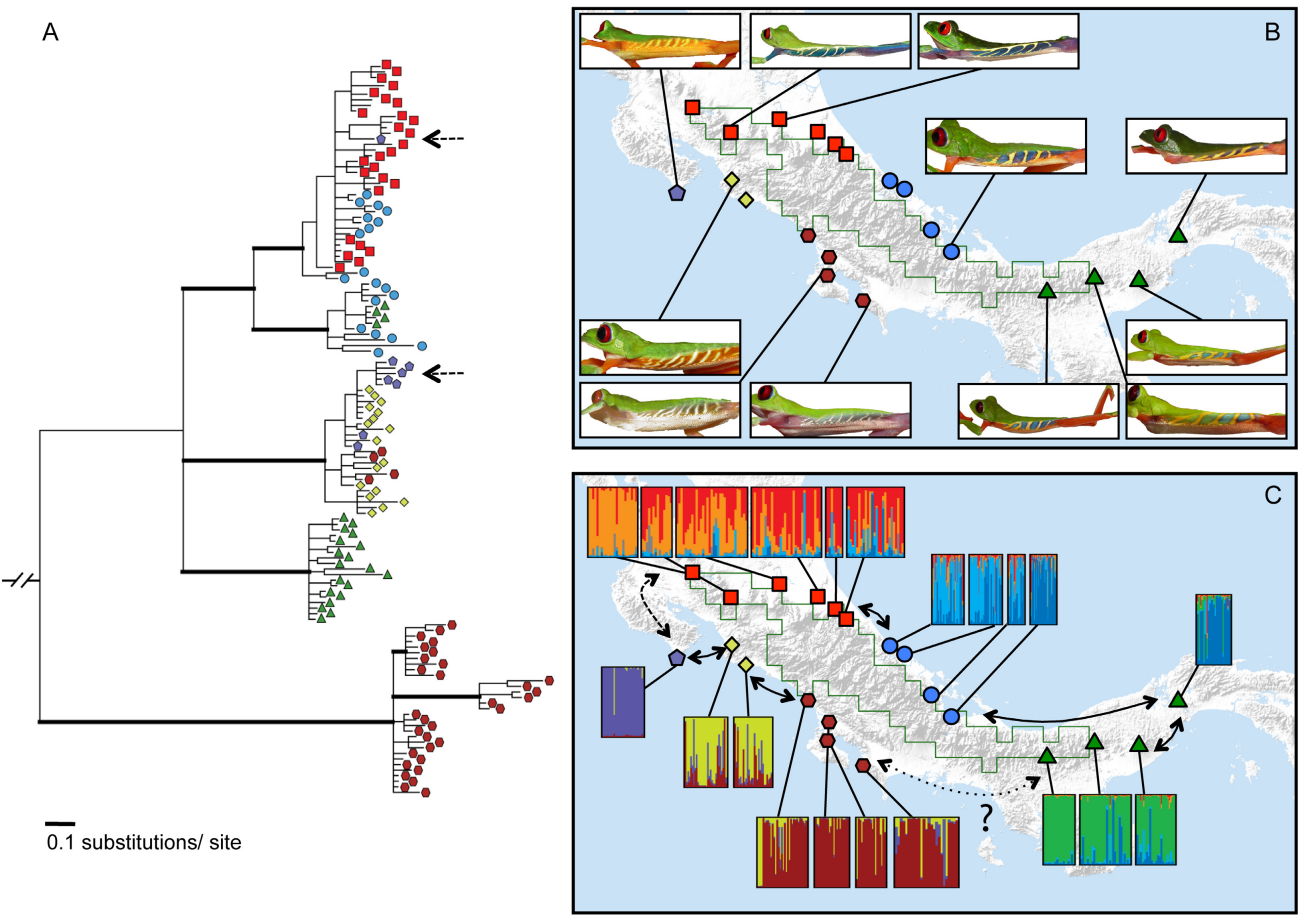
**Diversification of the red-eyed tree frog around a focal barrier in Costa Rica and Panama. A**: Phylogenetic relationships suggest diversification along the Atlantic and Pacific slopes of the barrier (major clades) followed by limited gene flow across parapatric boundaries (different symbols within clades). Currently allopatric populations in the northwestern portion of the species' range share mitochondrial haplotypes (dashed arrows) suggesting ancestral gene flow. The most genetically divergent taxa occur at the southeastern end of the known range (adapted from [22]). Bayesian consensus phylogram based on 1,149 base pairs of the NADH1 mitochondrial DNA gene fragment; rooted with the outgroup taxon *Agalychnis saltator*; thick branches are supported by > 0.95 posterior probability. **B**: Morphologic intergradation in flank stripe patterns and color pattern linking different ecomorphotypes [22,36]. **C**: Patterns of population structure suggesting ongoing gene flow as inferred from six microsatellite loci [22]. Bayesian assignment probabilities were inferred in the program Structure [37] identifying eight genetic demes with ongoing introgression among neighboring demes (solid arrows on map). The other two arrows represent current barriers to gene flow around the ring between the most phenotypically divergent taxa (dashed arrow), where mitochondrial data suggest ancestral gene flow, and the phylogenetically most divergent taxa (dotted arrow), where, based on current sampling, there is no such evidence and taxa seem to be separated by ecologically unsuitable habitat.

In Costa Rica and Panama, the candidate barrier is a mountain range (Cordillera de Talamanca) that separates the Pacific and Caribbean regions of the isthmus (Additional file 5). As a result of its particular topography, the Cordillera de Talamanca is surrounded by warmer habitats that are recognized as Isthmanian Pacific and Atlantic moist forest ecoregions [14]. These climatic conditions, plus the barrier, have shaped the distributions of many low elevation vertebrate and invertebrate species throughout the isthmus, and conditioned the diversification of lineages, which are currently recognized at various taxonomic ranks (see [20]) and often present evidence of morphologic and genetic intergradation (for example, *Heliconius *butterflies [21]). This diversification process is clearly illustrated by the red-eyed tree frog, *Agalychnis callidryas*, which forms a nearly complete ring distribution around the barrier (Figure 6). Extensive phenotypic (morphology, color pattern) and genotypic (mitochondrial and nuclear DNA) data have been collected to evaluate diversification patterns and their consequences for genetic interactions around the focal barrier [22,23]. In agreement with our topographic model prediction, neutral genetic data suggest that taxa have expanded around the central mountain, gradually diverging along the Pacific and Caribbean slopes (Figure 6A, [22,23]). Morphologic (Figure 6B) and genetic (Figure 6C) intergradation occurs between populations along either side of the mountain, suggesting ongoing gene flow around most of the ring distribution. Two notable exceptions occur between (i) the phenotypically most divergent populations in the northwestern portion of the range, where mitochondrial data suggest ancestral gene flow (Figure 6A, dashed arrow) but nuclear markers do not indicate ongoing connectivity (Figure 6C), and (ii) the phylogenetically most divergent populations in the southeastern portion of the range (Figure 6C, dotted arrow), where based on current sampling there is no such evidence of ancestral or ongoing gene flow. In order to corroborate the ring species hypothesis, evidence of ring closure with terminal reproductive isolation is needed, as demonstrated in other reference taxa (for example, [18]). Nevertheless, even without such evidence, published data demonstrate that the candidate barrier has strongly influenced adaptive and non-adaptive divergence among currently contiguous populations of *A. callidryas*, in the direction predicted for ring species.

**Figure 7:**
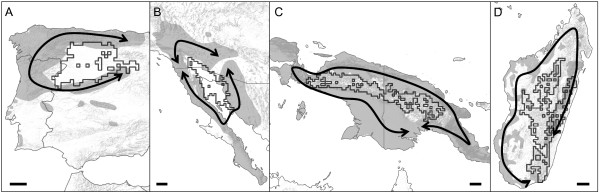
**Use of topographic ring model to identify candidate taxa for ring diversification around predicted barriers. A**: The monotypic species Schreiber's green lizard *Lacerta schreiberi*, Iberian Peninsula (southern Europe). **B**: The polytypic species rosy boa *Lichanura trivirgata*, Baja California Peninsula. **C**: The polytypic species little shrike-thrush *Colluricincla megarhyncha*, island of New Guinea. **D**: Closely related lemur species from the genus *Propithecus*, Madagascar. In all maps, predicted barriers are shown by the black outline of grid cells, documented ranges of taxa are shown as gray polygons, and expansions or colonizations around the barriers are shown by the black arrows, as inferred from genetic evidence (see Additional files 6, 7, 8 and 9). Black scale bars at the bottom of each map correspond to 100 k.

Additional barriers predicted by the topographic ring model are similarly surrounded by taxa showing continuous levels of divergence and exhibiting ill-defined taxonomic boundaries towards the terminus of a ring distribution - taxonomic irresolution characteristic of ring species (Figure 7, Additional files 6, 7, 8 and 9). In all of these taxa, the absence of clear and concordant genetic and morphologic discontinuities around their distribution has promoted ongoing discussions among taxonomists (for example, [24-26]), and such taxonomic irresolution is indeed expected when continuous stages of species formation are directly observed in nature.

## Conclusions

By identifying the geographic barriers around the world that are most likely to promote ring diversification, our model provides a formal and flexible approach to discovering new examples of geographic speciation across a diverse range of taxa and environments. Results of the model show that the topographic conditions required for ring speciation are rare when considered relative to all barriers on the planet, but remarkably common relative to the handful of known ring species. Model predictions further suggest that the majority of barriers that are topographically most likely to provide new examples of ring speciation occur in under-studied parts of the world. Although model predictions are presently limited by the few clear examples of ring species, the discovery of new ring taxa will allow iterations of this same model with numerous and biologically diverse taxa. New applications and parameterizations of the model using topography and other environmental gradients will create additional opportunities to study geographic divergence towards the formation of new species in nature, especially across taxa with different population biologies and that diversify at different spatial scales. As Mayr ([2], p. 182) stated, "overlapping rings (that is, ring species) are disturbing to the orderly mind of the cataloguing systematist, but they are welcome to the student of speciation". We predict that taxa associated with focal barriers emerging from our model will express patterns of clinal differentiation in a direction towards species formation, thus illustrating examples of taxonomic irresolution. Irrespective of whether terminal taxa are above or below species level divergence, these examples allow us to identify the areas where additional evolutionary processes necessary for ring divergence can take place (that is, adaptive divergence) and promote diversification in nature.

## Methods

### Reference ring taxa

For purposes of training our model, we selected four reference taxa described in the literature as ring species: the salamander *Ensatina eschscholtzii *[19]; the tree *Acacia karroo *[27,28]; the bird *Phylloscopus trochiloides *[12]; and the bird species complex *Larus *(sp. *argentatus *and *fuscus*) [2]. Reference ring taxa are also reviewed by Irwin *et al. *[6]. Importantly, due to recent advances in new taxonomic tools and criteria, these are not necessarily all recognized unambiguously as 'ring species', but do in all cases constitute taxa that are evolving under ring models of divergence - that is, 'ring taxa' that express continuous levels of differentiation with terminal forms above or below species level. Further, we restricted our analysis to these four taxa because they (i) represent true circular overlaps around distinct physical geographic barriers, *sensu *Mayr [8], as opposed to other ring systems produced by rare dispersal events; and (ii) have well described distributions with maps and extensive text-based descriptions that enabled us to extract the reference barriers from our model.

### Topographic model

Rather than modeling ring distributions of species, our predictive model targets the geographic barriers that are topographically similar to barriers associated with taxa that are considered ring species. We focus on the geographic barrier because it is a core feature of all well-documented ring species, thus enabling us to make predictions about candidate rings across taxa and environments. Our model involves four steps (Figure 1): (i) selecting the focal environmental gradient, (ii) deriving the rate of change in the gradient, (iii) extracting all barriers and calculating summary statistics for traits relevant to geographic species formation, and (iv) analyzing the traits in multivariate space. We describe these steps in detail below.

#### Focal gradient

Step 1 in Figure 1A. We selected elevation as our focal gradient because it is often correlated with other major environmental gradients that more proximately determine barriers to species distribution [29], and high-quality elevation data are available from multiple sources for the entire globe. Combined, these features of elevation enabled to us build a topographic model that could be reliably generalized to all environments. The model may be parameterized using other environmental gradients to address more targeted questions in specific taxa or geographies. Elevation data were obtained from the National Geophysical Data Center, National Oceanic and Atmospheric Administration's ETOPO1 One Arc-Minute Global Relief Model [30]. We selected the bedrock layer in order to define elevations irrespective of spatiotemporally fluctuating ice sheets and glaciers.

#### Rate of gradient change

Step 2 in Figure 1B. Our model was based on the rate of change in elevation (topographic slope) because this variable is associated with ecotones at landscape to biome scales [13] in all terrestrial, aquatic and marine environments around the world. In turn, ecotones, and associated ecoregions, are strong predictors of species' distributions [14]. We computed slope on the original ETOPO1 bedrock raster and then resampled it using bilinear interpolation to 10 arc minute. This decision was made to provide a spatial resolution that yielded a biologically reasonable minimum barrier size. Since the area of a 10 arc minute cell decreases with increasing latitude, minimum barrier size ranged from 344.2 km^2 ^at the equator to 1.5 km^2 ^at the poles (we accounted for this in our summary statistic calculations using spherical trigonometry, see below). But because the range sizes of taxa tend to decrease with increasing latitude [31], we considered this to be a biologically reasonable minimum barrier size for low-dispersing or recently diverged taxa, irrespective of their latitude of origin. Furthermore, 10 arc minute resolution was determined through an initial sensitivity analysis to most accurately approximate the range of reference barriers.

#### Identifying barriers

Step 3 in Figure 1C. Empirical field-based studies have described ring species as taxa that diversify around a geographic barrier, which could be as small as the Central Valley of California or as large as Central Asia [6]. However, because these studies did not require a definition of what is a barrier, the geographic barriers associated with classical ring systems could not be explicitly compared to one another, or to other barriers on the planet. We formally defined barriers in our model as geographically contiguous blocks of grid cells that, at the 10 arc minute resolution of our analysis, had the potential to physically separate two or more taxa. Throughout we refer to these geographically contiguous barriers as being 'cohesive' because they are comprised of cells that stick together. Although slope is a continuous variable, calculations of topographic traits required discrete barriers (that is, groups of cells that constituted a cohesive barrier). We first reclassified into separate sets of grids all grid cells that were either greater than or less than or equal to a certain slope threshold. The resulting sets of cells that met the conditional statement on each grid effectively defined our candidate binary barriers for that threshold. Slope thresholds were allowed to vary from 1 to 87 (maximum observed) degrees, in increments of 1 degree, in order to bracket the complete range of barriers that species could be responding to. The biological rationale for thresholding slope in this fashion relates to the two main conditions that enable ecotones [13,32]: (i) steep physical environmental gradients that directly affect key ecological processes and the distribution of organisms, and (ii) gradual physical environmental gradients where threshold or nonlinear responses cause changes in ecosystem dynamics and the distributions of dominant species. Hence, the ecotones that define geographic barriers in our model may be important for taxa irrespective of whether the slope is steep or shallow. For each slope threshold, in determining how to group sets of cells into discrete barriers, we further defined barriers as geographically cohesive blocks of grid cells under one-cell rook chess moves in the four cardinal directions. Cell blocks were then indexed sequentially on a sphere in order to eliminate edge-effects at poles and the International Date Line.

#### Topographic summary statistics

Table 1. We selected a total of six biologically informative summary statistics that collectively capture the size, position and permeability of candidate barriers: area, perimeter, maximum latitudinal range (controlling for longitude, max latitude minus min latitude), mean distance from equator (based on the absolute value of the centroid of each barrier), perimeter-to-area ratio, and fragmentation. All area and distance summary statistics were computed using spherical trigonometry [33] to eliminate geographic bias in distortion introduced by imposing planar projections, and also to enable a comprehensible analysis of Polar Regions.

Fragmentation was evaluated separately for all candidate barriers in two steps. First, beginning with the slope threshold yielding the largest and globally most inclusive candidate barriers (1 degree for grids greater than each slope threshold and 87 degrees for grids less than or equal to each slope threshold), we determined the number of sequential slope thresholds (*x*) that maintained the starting barrier unfractured in smaller form as spatially contiguous blocks of cells. We also used barriers identified in *x *to derive mean estimates of the other five summary statistics that were computed using spherical trigonometry. Because mountains and valleys serve as barriers to species' distributions in similar ways, we did not distinguish between the two types of barrier inflection, and thus combined them for purposes of analysis. This process effectively reduced the number of redundant barriers (that is, barriers preserved across multiple slope thresholds) from 7,045,548 to 952,147. In other words, our inclusion of fragmentation allowed us to eliminate 6,093,401 spatially redundant or overlapping barriers that were originally extracted from applying the slope thresholds. Second, we calculated fragmentation as 1 - (*x*/*a*), where *a *= the maximum number of slope thresholds possible, 87 for our analysis.

#### Principal component analysis

Step 4 in Figure 1D. We used the summary statistics to compare the candidate barriers in a principal component analysis (PCA). Log transformations were applied to area, distance, and shape summary statistics; an arcsin transformation was used on fragmentation. The multivariate PCA space (barrier-space) was used to identify candidate barriers that were topographically equivalent to those known to be associated with ring species (see below). We provide our complete model results (data deposited in the Dryad Repository: http://dx.doi.org/10.5061/dryad.5856q415) so that future studies can evaluate new hypotheses of barriers that may be promoting ring divergence.

### Identification of reference and candidate barriers

Following development of the topographic model, we identified the barriers associated with our reference taxa ('reference barriers'), and also the other barriers from around the world that were topographically similar to the reference barriers ('candidate barriers').

#### Reference barriers

We identified reference barriers by visually inspecting which topographic barriers from the model were circumscribed by the distributions of reference taxa. Data on the distributions of reference taxa were obtained from a combination of georeferenced point localities and range maps and included the following sources: *Ensatina *[18], *Acacia *[27], *Phylloscopous *[34], and *Larus *[34]. We then used principal components 1 and 2 (PC1, PC2) to extract the locations in multidimensional space of the reference barriers associated with our reference ring taxa. We determined that both *Ensatina *and *Acacia *encircled single barriers ('cohesive barriers'), while *Larus *and *Phylloscopus *each encircled clusters of two or three cohesive barriers in close geographic proximity (thus forming 'composite barriers'). For purposes of describing the geography of each cohesive and composite reference barrier, we identified the common names of the topographic features from multiple world and regional maps.

#### Candidate barriers

Because geographic species formation depends on the interaction between the 'scale' of the organism and the spatial scale of the barrier associated with it, both the population biology and history of reference and candidate taxa need to be considered when evaluating candidate barriers. Thus, we identified candidate barriers separately with respect to the reference taxa and associated barriers. Because reference barriers were discovered to be either cohesive or composite, we further identified candidate barriers according to two methods.

##### Candidate cohesive barriers (*Ensatina *and *Acacia*)

Candidate cohesive barriers are represented in the model by other individual barriers that are topographically similar to the reference barriers in multidimensional barrier space. For this method, we identified candidate cohesive barriers as the 100 nearest neighbors (Euclidean distance) to each reference barrier in the PCA. Yet, other criteria could alternatively be used to define similarity (for example, a Euclidean buffer around reference barriers). For each reference taxon and associated barrier, we mapped the candidates back into geographic space and summed across barriers to detect possible spatial overlap of topographically similar candidates that were not consolidated using our estimate of fragmentation.

##### Candidate composite barriers (*Larus *and *Phylloscopus*)

Composite barriers are represented in the model by groups of other individual cohesive barriers. In addition to the summary statistics characteristic of all barriers, reference composite barriers are described by a particular combination of individual barriers with four criteria: (i) number of barriers, (ii) total area, (iii) geographic proximity to one another, and (iv) the same inflection. Individual barriers were first queried by identifying the 100 nearest neighbors (Euclidean distance) relative to each of the five reference barriers. Because these barriers clustered with respect to both reference bird taxa, we used a minimum convex polygon around the 500 total nearest neighbors to identify 1,380 candidate barriers. As performed for *Ensatina *and *Acacia*, we mapped the candidates back into geographic space and summed across barriers to detect spatial overlap of topographically similar candidates that were not consolidated using our estimate of fragmentation. We then queried for candidate composite barriers separately in *Larus *and *Phylloscopus *using the four criteria above, with similarity thresholds set to 5%. In the case of the Arctic Ocean, which was comprised of three individual reference barriers, we employed a two-barrier approximation because 90% of the total area was explained by two reference barriers (the Canada and Amundsen-Nansen Basins).

## Abbreviations

PC1 and PC2: principal components; PCA: principal component analysis.

## Competing interests

The authors declare that they have no competing interests.

## Authors' contributions

WBM and RJP conceptualized the topographic ring model. WBM developed and implemented the model and performed statistical analysis. WBM, RJP and DBW interpreted the results and helped to draft the manuscript. All authors read and approved the final manuscript.

## Supplementary Material

Additional file 1 **Examples of predicted barriers and how they align with associated ecoregions or major oceanic features**. Barriers are shown by red polygons, ecoregions by black outlines, and global elevations by the shaded topography. **A**: Zambezian flooded grasslands. **B**: Sichuan basin broadleaf evergreen forests. **C**: Kuh Rud and Eastern Iran montane woodlands. **D**: Eastern Guinean forests. **E**: Sea of Azov (SE Ukraine). **F**: southern portions of the Chilean matorral. **G**: multiple ecoregions in the Andes. **H**: multiple ecoregions on the Arabian Peninsula. **I**: South Equatorial Current. **J**: Falkland Current. **K**: Alaska Current. **L**: Bering Sea (North is oriented down). Click here for file

Additional file 2 **Additional ring-distributed taxa surrounding reference barriers. A**: The bird species complex *Alauda *(sp. *arvensis *and *gulgula*): Central Asia. **B**: The bird species *Parus major*: Central Asia. **C**: The bird species complex *Charadrius *(sp. *hiaticula *and *semipalmatus*): Arctic Ocean. Barriers are shown by the red polygons, species' distributions by the black points and gray polygons (*Charadrius*), and global elevations by the shaded topography. Numbers correspond to individual barriers identified in the PCA. Click here for file

Additional file 3 **Principal component analysis**. Principal component scores (PC1, PC2) for each summary statistic included in the topographic ring model. Click here for file

Additional file 4 **Barrier permeability as measured by fragmentation and shape**. Left: barrier permeability (fragmentation) vs. size (area). Right: barrier permeability (shape as measured by the perimeter-to-area ratio) vs. size (area). Vertical lines identify barriers that are 50,000 km^2^. Gray points identify all barriers on the planet, as defined by the topographic model. Green points identify barriers where fragmentation < 1. Numbers correspond to barriers associated with known ring taxa: (1) *Ensatina*: Central Valley, California, USA; (2) *Acacia*: Drakensberg Massif, South Africa; (3) *Larus*: Makarov Basin, Arctic Ocean; (4) *Phylloscopus*: Tibetan Plateau, Central Asia; (5) *Phylloscopus*: Takla Maka and Gobi Deserts, Central Asia; (6) *Larus*: Amundsen and Nansen Basins, Arctic Ocean; (7) *Larus*: Canada Basin, Arctic Ocean. Smaller perimeter-to-area ratios describe barriers that are more circular and compact. Click here for file

Additional file 5 **Use of the topographic ring model to identify candidate taxa for ring diversification around a focal barrier in Costa Rica and Panama that is topographically similar to the reference barrier for the Central Valley (California, USA), which has promoted ring diversification in a salamander, *Ensatina eschscholtzii***. **A**: The focal barrier is a long-standing geographic feature known as the Cordillera de Talamanca. **B**: As a result of its particular topography, the mountainous barrier is surrounded at lower elevations by higher temperatures. **C**: In part due to these temperature gradients, the predicted barrier is considered a distinct ecoregion (Talamancan Montane Forests) that is surrounded by other distinct ecoregions, which form a ring distribution. **D**: These climatic and ecoregional conditions have shaped the distribution of many species, including the red-eyed tree frog, *Agalychnis callidryas*. Click here for file

Additional file 6 **Use of the topographic ring model to identify candidate taxa for ring diversification around a focal barrier in the Iberian Peninsula (southern Europe) that is topographically similar to the reference barrier for the Drakensberg Massif (South Africa), which has promoted ring diversification in a tree species, *Acacia karroo***. Extensive field-based studies in Iberia - particularly in reptiles and amphibians - have generated considerable distributional and phylogeographic data that can be used to evaluate whether the focal barrier has promoted continuous levels of differentiation typical of ring divergence. The focal barrier (top panel, map) is a long-standing geographic barrier for terrestrial organisms, serving as a steep ecotone between the main climatic regions of Iberia. As a result of its particular topography, a central arid and warmer plateau is surrounded by moister and colder habitat. These climatic conditions have shaped the distribution of many Atlantic species on the peninsula, including the fire salamanders *Salmandra salamandra*, and also Schreiber's green lizard *Lacerta schreiberi*, which forms a nearly complete ring distribution around the barrier (map). Extensive genetic data (in both mitochondrial and nuclear DNA) have been collected to reconstruct its phylogeographic history. In agreement with our model prediction, multi-locus data suggest that the focal barrier has strongly influenced non-adaptive divergence among currently contiguous populations of *L. schreiberi*, showing evidence of continuous levels of genetic differentiation around the barrier and no evidence of historical gene flow across it (bottom panel, phylogenetic network; thick branches are supported by > 0.95 posterior probability). Although the species in this example lacks terminal overlap, it illustrates how the topographic ring model may be used to properly identify and evaluate new instances of ring diversification. Click here for file

Additional file 7 **Use of the topographic ring model to identify candidate taxa for ring diversification around a focal barrier near the Baja California Peninsula (USA and Mexico) that is topographically similar to the reference barrier for the Drakensberg Massif (South Africa), which has promoted ring diversification in a tree species, *Acacia karroo***. The focal barrier (left panel, map) is a low-lying topographic depression located at the land-sea interface in the northern Sea of Cortez. As a result of its particular topography, the barrier has promoted diversification in a number of terrestrial taxa, including *Hypsiglena *nightsnakes and the rosy boa *Lichanura trivirgata*. In *L. trivirgata*, mitochondrial data have been collected to reconstruct its phylogeographic history. In agreement with our model prediction, these data suggest that the focal barrier has strongly influenced non-adaptive divergence among mostly contiguous subspecies of *L. trivirgata*, showing evidence of continuous levels of genetic differentiation along either side of the barrier (right panel, phylogenetic network; thick branches are supported by > 0.95 posterior probability). Closure of the ring distribution may occur in the northwest, between two deeply divergent lineages within the subspecies *roseofusca *(symbolized by circles and hexagons). Click here for file

Additional file 8 **Use of the topographic ring model to identify candidate taxa for ring diversification around a focal barrier on the island of New Guinea that is topographically similar to the reference barrier for the Central Valley (California, USA), which has promoted ring diversification in a salamander, *Ensatina eschscholtzii***. The focal barrier (upper right panel, map) is a mountain forest ecoregion that is surrounded at lower elevations by warmer and generally drier ecoregions and basins. This distribution of contrasting bioclimates is hypothesized to have promoted diversification in a number of bird taxa, including *Pitohui, Tanysiptera *kingfishers, *Aegotheles *owlet-nightjars, and the little shrike-thrush *Colluricincla megarhyncha*. All of these taxa are monophyletic and have diversified around the barrier, reaching different stages of divergence. This diversification is especially well illustrated by *C. megarhyncha*, where mitochondrial data have been collected to reconstruct its phylogeographic history. In agreement with our model prediction, these data suggest that the focal barrier has strongly influenced non-adaptive divergence among mostly contiguous subspecies of *C. megarhyncha*, showing evidence of continuous levels of genetic differentiation along either side of the barrier (left panel, phylogenetic network; numbers report numbers of site or base pair substitutions between haplotypes). Additionally, plots of genetic vs. geographic distance (lower right panel, plot) reveal significant isolation by distance around the barrier, but not across it, suggesting that this is an important barrier to colonization and gene flow. Although it is unclear whether there is terminal overlap at the southern end of the ring distribution, this example illustrates how the topographic ring model may be used to properly identify and evaluate new instances of ring diversification. Click here for file

Additional file 9 **Use of the topographic ring model to identify candidate taxa for ring diversification around a focal barrier in Madagascar that is topographically "in between" (**Figure 3**) reference barriers for the Drakensberg Massif (South Africa), which has promoted ring diversification in a tree species, *Acacia karroo*, and the Tibetan Plateau (Central Asia), which has promoted ring diversification in a bird species, *Phylloscopus trochiloides***. The focal barrier (right panel, map) is a mountainous subhumid bioclimatic zone surrounded at lower elevations by humid (east) and subarid/dry (west) zones. This distribution of contrasting bioclimates is hypothesized to have promoted diversification in amphibians, reptiles, and lemurs, some of which form either complete or nearly complete ring distributions around the barrier. In *Propithecus *lemurs, mitochondrial data have been collected to reconstruct its phylogeographic history. In agreement with our model prediction, these data suggest that the focal barrier has strongly influenced non-adaptive divergence among mostly contiguous species of *Propithecus*, showing evidence of continuous levels of genetic differentiation (from north to south) along either side of the barrier (left panel, phylogenetic tree; thick branches are supported by > 0.95 posterior probability). Although there appears to be no overlap of terminal taxa in the south, this example illustrates how the topographic ring model may be used to properly identify and evaluate new instances of ring diversification. Click here for file
