# Supplementary material for: Ring distributions leading to species formation: a global topographic analysis of geographic barriers associated with ring species
Source: BMC Biol. 2012 Mar 12;10:20. doi: 10.1186/1741-7007-10-20 (PMC3320551; doi:10.1186/1741-7007-10-20)
Supplement: Additional file 1 — Examples of predicted barriers and how they align with associated ecoregions or major oceanic features. Barriers are shown by red polygons, ecoregions by black outlines, and global elevations by the shaded topography. A: Zambezian flooded grasslands. B: Sichuan basin broadleaf evergreen forests. C: Kuh Rud and Eastern Iran montane woodlands. D: Eastern Guinean forests. E: Sea of Azov (SE Ukraine). F: southern portions of the Chilean matorral. G: multiple ecoregions in the Andes. H: multiple ecoregions on the Arabian Peninsula. I: South Equatorial Current. J: Falkland Current. K: Alaska Current. L: Bering Sea (North is oriented down). [file 1741-7007-10-20-S1.PDF]

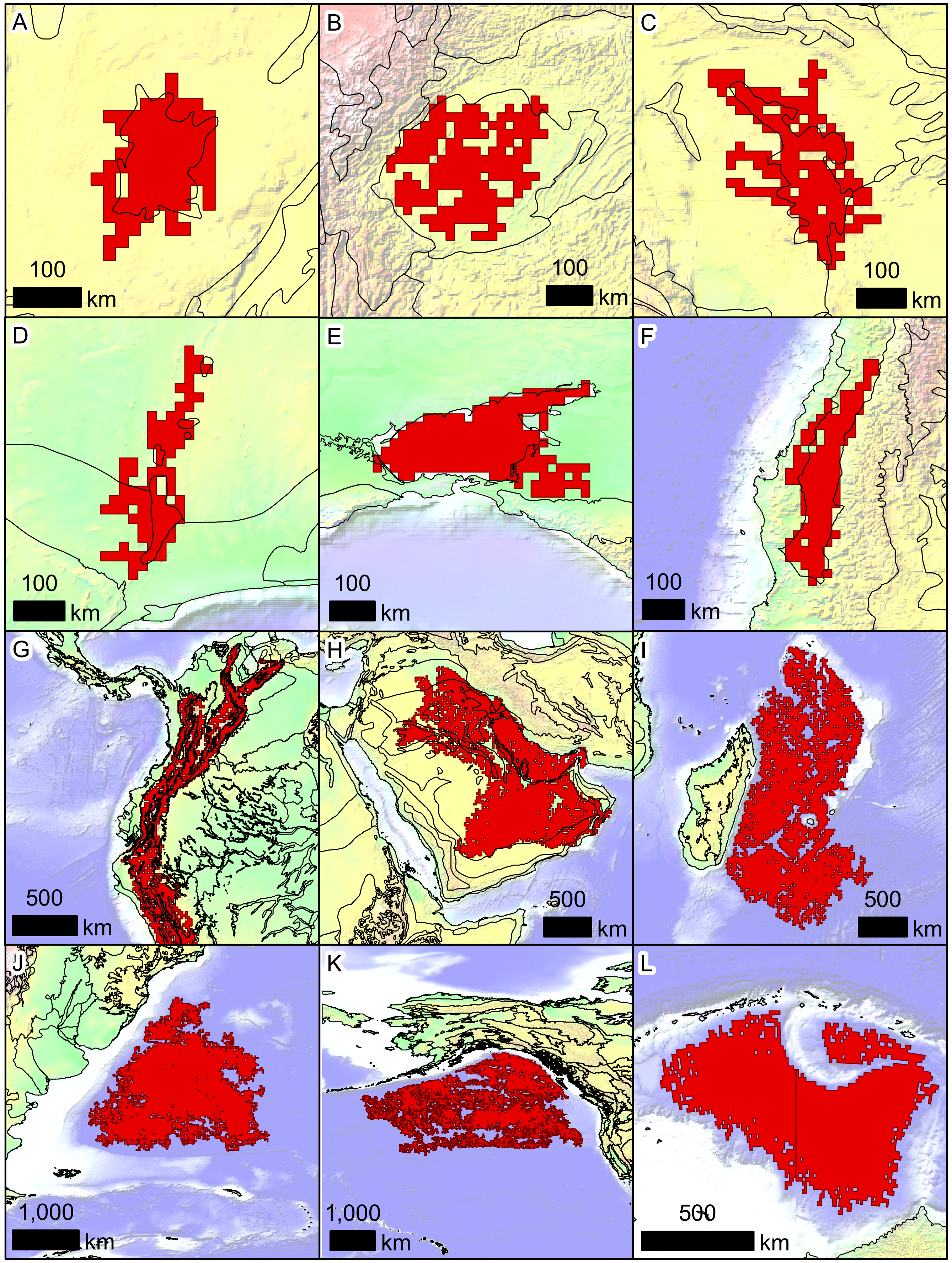

Examples of predicted barriers and how they align with associated ecoregions or major oceanic features. Barriers are shown by red polygons, ecoregions [1] by black outlines, and global elevations by the shaded topography. A: Zambezian flooded grasslands. B: Sichuan basin broadleaf evergreen forests. C: Kuh Rud and Eastern Iran montane woodlands. D: Eastern Guinean forests. E: Sea of Azov (SE Ukraine). F: southern portions of the Chilean matorral. G: multiple ecoregions in the Andes. H: multiple ecoregions on the Arabian Peninsula. I: South Equatorial Current. J: Falkland Current. K: Alaska Current. L: Bering Sea (North is oriented down).

References

1. Olson DM, Dinerstein E, Wikramanayake ED, Burgess ND, Powell GVN, Underwood EC, D'Amico JA, Itoua I, Strand HE, Morrison JC, Loucks CJ, Allnutt TF, Ricketts TH, Kura Y, Lamoreux JF, Wettengel WW, Hedao P, Kassem KR: **Terrestrial ecoregions of the world: a new map of life on earth**. *BioScience* 2001, **51**:933-938.
