# Supplementary material for: Ring distributions leading to species formation: a global topographic analysis of geographic barriers associated with ring species
Source: BMC Biol. 2012 Mar 12;10:20. doi: 10.1186/1741-7007-10-20 (PMC3320551; doi:10.1186/1741-7007-10-20)
Supplement: Additional file 2 — Additional ring-distributed taxa surrounding reference barriers. A: The bird species complex Alauda (sp. arvensis and gulgula): Central Asia. B: The bird species Parus major: Central Asia. C: The bird species complex Charadrius (sp. hiaticula and semipalmatus): Arctic Ocean. Barriers are shown by the red polygons, species' distributions by the black points and gray polygons (Charadrius), and global elevations by the shaded topography. Numbers correspond to individual barriers identified in the PCA. [file 1741-7007-10-20-S2.PDF]

Monahan, Pereira, & Wake: Ring Distributions Leading to Species Formation
Additional file 2

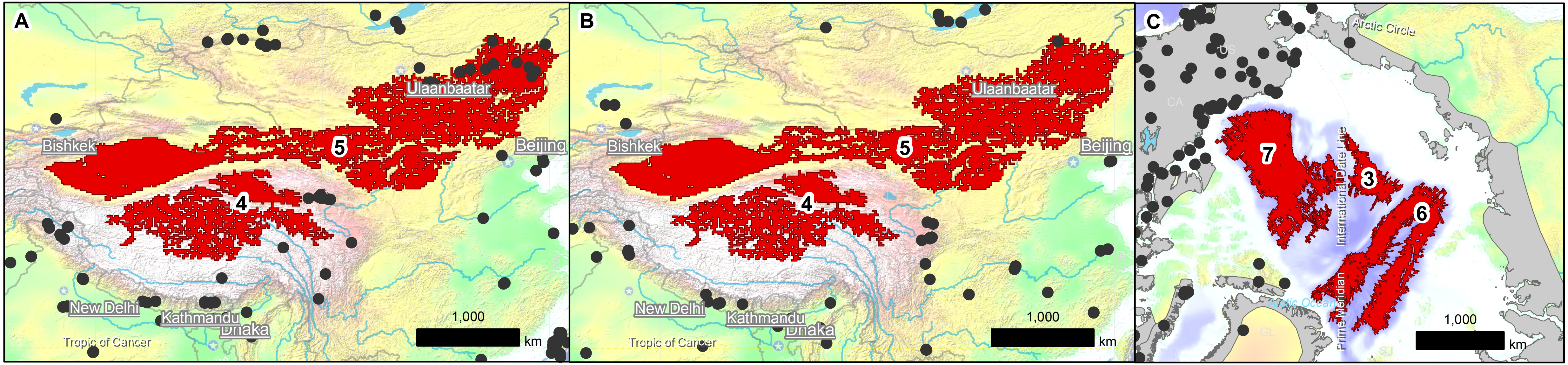

Additional ring-distributed taxa surrounding reference barriers. A: The bird species complex *Alauda* (sp. *arvensis* and *gulgula*) [1,2]: Central Asia. B: The bird species *Parus major* [2,3]: Central Asia. C: The bird species complex *Charadrius* (sp. *hiaticula* and *semipalmatus*) [2,4]: Arctic Ocean. Ring taxa also reviewed by Irwin et al. [5]. Barriers are shown by the red polygons, species' distributions by the black points [6] and gray polygons (*Charadrius*; [7]), and global elevations by the shaded topography. Numbers correspond to individual barriers identified in the PCA.

References

1. Vaurie C: **A study of Asiatic larks.** *Bull Am Nat Hist* 1951, **97**:431-526.
2. Mayr E: *Animal Species and Evolution*. Cambridge, MA: Belknap Press; 1963.
3. Mayr E: *Systematics and the Origin of Species, from the Viewpoint of a Zoologist*. Cambridge, MA: Harvard University Press; 1942.
4. Bock WJ: **The status of the semipalmated plover.** *Auk* 1959, **76**:98-100.
5. Irwin DE, Irwin JH, Price TD: **Ring species as bridges between microevolution and speciation.** *Genetica* 2001, **112-113**:223-243.
6. Ornithology Information System (ORNIS): Data were obtained from all contributing institutions with georeferenced localities on 25 September 2001. http://www.ornisnet.org.
7. BirdLife International and NatureServe: *Bird species distribution maps of the world*. Cambridge, UK and Arlington, USA: BirdLife International and NatureServe; 2011.
