# Supplementary material for: Ring distributions leading to species formation: a global topographic analysis of geographic barriers associated with ring species
Source: BMC Biol. 2012 Mar 12;10:20. doi: 10.1186/1741-7007-10-20 (PMC3320551; doi:10.1186/1741-7007-10-20)
Supplement: Additional file 3 — Principal component analysis. Principal component scores (PC1, PC2) for each summary statistic included in the topographic ring model. [file 1741-7007-10-20-S3.PDF]

Monahan, Pereira, & Wake:
Ring Distributions Leading to Species Formation

Additional file 3. Principal component scores (PC1, PC2) for each summary statistic included in the topographic ring model.

Summary Statistic	PC1	PC2
Area	-0.508	0.026
Perimeter	-0.500	-0.083
Latitudinal extent	-0.481	-0.167
Perimeter-to-area ratio	0.459	-0.246
Mean distance from equator	0.125	-0.778
Fragmentation	-0.185	-0.547
