# Supplementary material for: Ring distributions leading to species formation: a global topographic analysis of geographic barriers associated with ring species
Source: BMC Biol. 2012 Mar 12;10:20. doi: 10.1186/1741-7007-10-20 (PMC3320551; doi:10.1186/1741-7007-10-20)
Supplement: Additional file 4 — Barrier permeability as measured by fragmentation and shape. Left: barrier permeability (fragmentation) vs. size (area). Right: barrier permeability (shape as measured by the perimeter-to-area ratio) vs. size (area). Vertical lines identify barriers that are 50,000 km2. Gray points identify all barriers on the planet, as defined by the topographic model. Green points identify barriers where fragmentation < 1. Numbers correspond to barriers associated with known ring taxa: (1) Ensatina: Central Valley, California, USA; (2) Acacia: Drakensberg Massif, South Africa; (3) Larus: Makarov Basin, Arctic Ocean; (4) Phylloscopus: Tibetan Plateau, Central Asia; (5) Phylloscopus: Takla Maka and Gobi Deserts, Central Asia; (6) Larus: Amundsen and Nansen Basins, Arctic Ocean; (7) Larus: Canada Basin, Arctic Ocean. Smaller perimeter-to-area ratios describe barriers that are more circular and compact. [file 1741-7007-10-20-S4.PDF]

Monahan, Pereira, & Wake: Ring Distributions Leading to Species Formation
Additional file 4

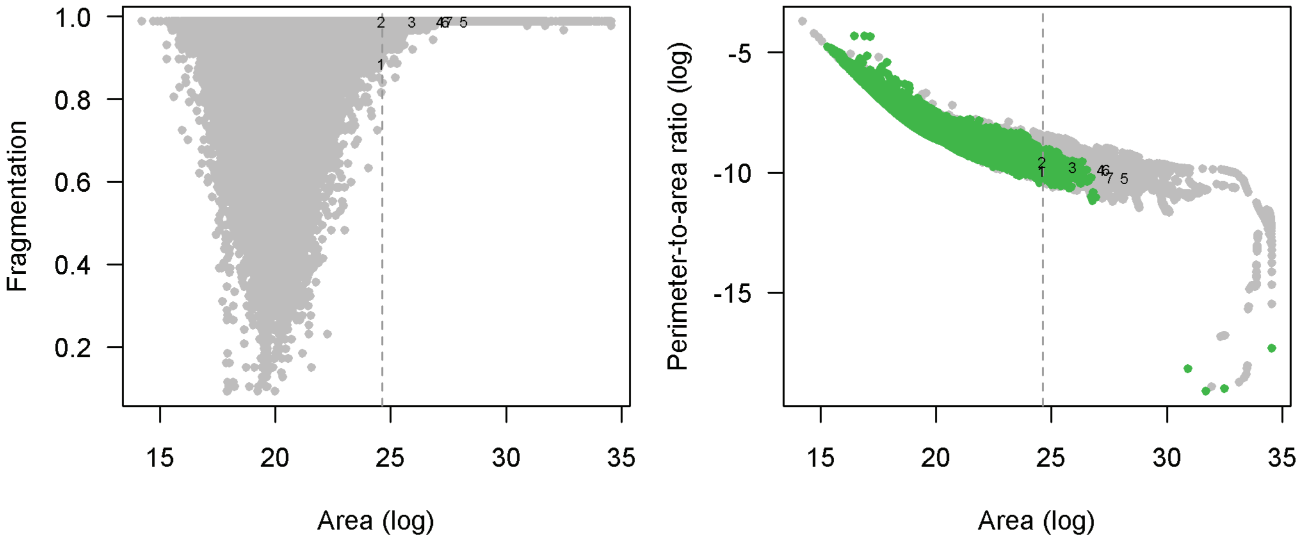

Barrier permeability as measured by fragmentation and shape. Left: barrier permeability (fragmentation) vs. size (area). Right: barrier permeability (shape as measured by the perimeter-to-area ratio) vs. size (area). Vertical lines identify barriers that are 50,000 km^2. Gray points identify all barriers on the planet, as defined by the topographic model. Green points identify barriers where fragmentation < 1. Numbers correspond to barriers associated with known ring taxa: (1) *Ensatina*: Central Valley, California, USA; (2) *Acacia*: Drakensberg Massif, South Africa; (3) *Larus*: Makarov Basin, Arctic Ocean; (4) *Phylloscopus*: Tibetan Plateau, Central Asia; (5) *Phylloscopus*: Takla Maka and Gobi Deserts, Central Asia; (6) *Larus*: Amundsen and Nansen Basins, Arctic Ocean; (7) *Larus*: Canada Basin, Arctic Ocean. Smaller perimeter-to-area ratios describe barriers that are more circular and compact.
