# Supplementary material for: Ring distributions leading to species formation: a global topographic analysis of geographic barriers associated with ring species
Source: BMC Biol. 2012 Mar 12;10:20. doi: 10.1186/1741-7007-10-20 (PMC3320551; doi:10.1186/1741-7007-10-20)
Supplement: Additional file 5 — Use of the topographic ring model to identify candidate taxa for ring diversification around a focal barrier in Costa Rica and Panama that is topographically similar to the reference barrier for the Central Valley (California, USA), which has promoted ring diversification in a salamander, Ensatina eschscholtzii. A: The focal barrier is a long-standing geographic feature known as the Cordillera de Talamanca. B: As a result of its particular topography, the mountainous barrier is surrounded at lower elevations by higher temperatures. C: In part due to these temperature gradients, the predicted barrier is considered a distinct ecoregion (Talamancan Montane Forests) that is surrounded by other distinct ecoregions, which form a ring distribution. D: These climatic and ecoregional conditions have shaped the distribution of many species, including the red-eyed tree frog, Agalychnis callidryas. [file 1741-7007-10-20-S5.PDF]

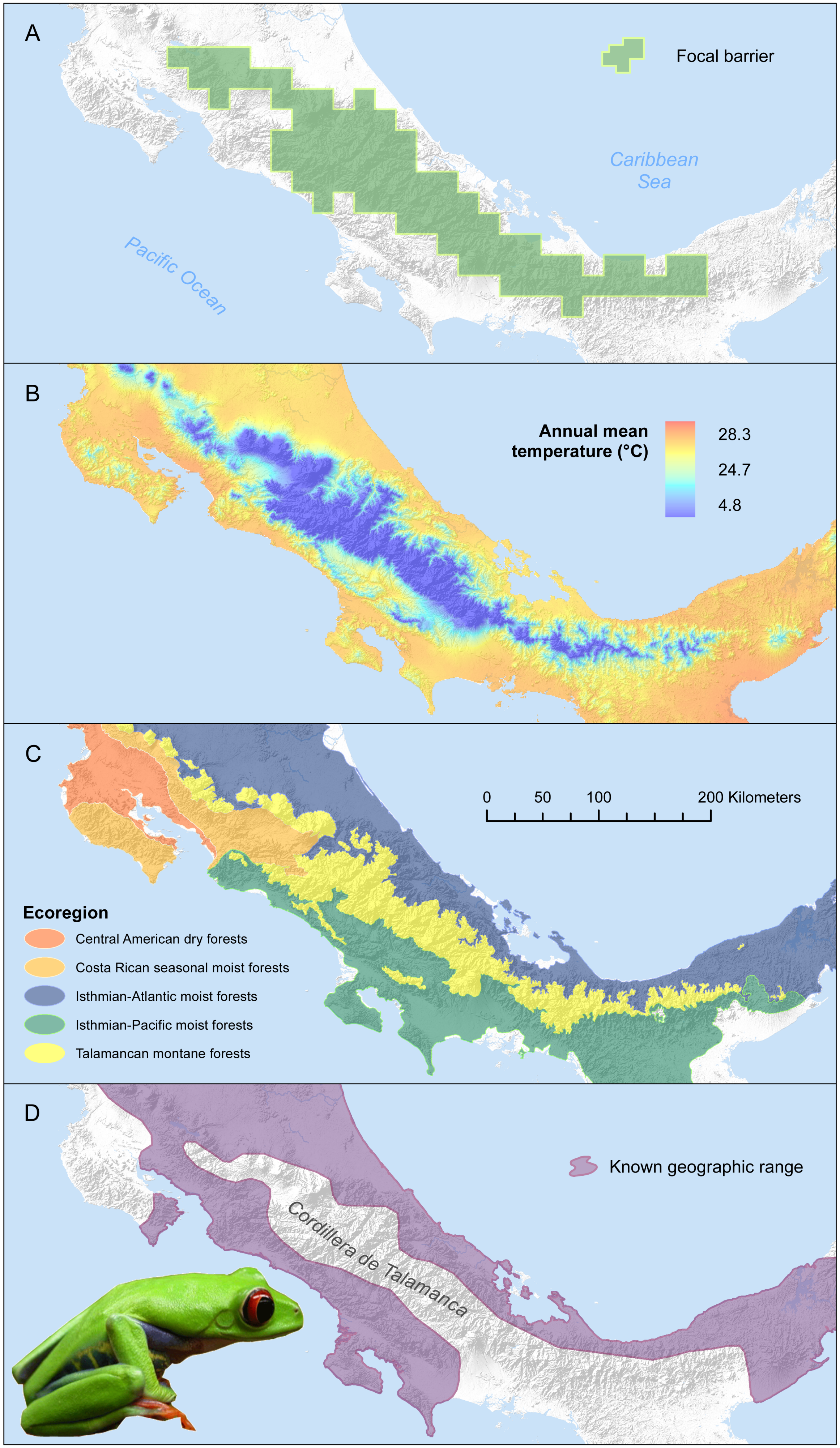

Use of the topographic ring model to identify candidate taxa for ring diversification around a focal barrier in Costa Rica and Panama that is topographically similar to the reference barrier for the Central Valley (California, USA), which has promoted ring diversification in a salamander, *Ensatina eschscholtzii* [1]. A: The focal barrier is a long-standing geographic feature known as the Cordillera de Talamanca. B: As a result of its particular topography, the mountainous barrier is surrounded at lower elevations by higher temperatures [2]. C: In part due to these temperature gradients, the predicted barrier is considered a distinct ecoregion (Talamancan Montane Forests) that is surrounded by other distinct ecoregions [3], which form a ring distribution. D: These climatic and ecoregional conditions have shaped the distribution of many species, including the red-eyed tree frog, *Agalychnis callidryas* [4]. Photo of *A. callidryas* provided by Jeanne Robertson.

References

1. Wake DB: **Incipient species formation in salamanders of the *Ensatina complex*.** *Proc Natl Acad Sci USA* 1997, **94**:7761-7767.
2. Hijmans RJ, Cameron SE, Parra JL, Jones PG, Jarvis A: **Very high resolution interpolated climate surfaces for global land areas.** *Int J Climatol* 2005, **25**:1965-1978.
3. Olson DM, Dinerstein E, Wikramanayake ED, Burgess ND, Powell GVN, Underwood EC, D'Amico JA, Itoua I, Strand HE, Morrison JC, Loucks CJ, Allnutt TF, Ricketts TH, Kura Y, Lamoreux JF, Wettengel WW, Hedao P, Kassem KR: **Terrestrial ecoregions of the world: a new map of life on earth**. *BioScience* 2001, **51**:933-938.
4. IUCN: **IUCN Red List of Threatened Species, version 2009.1.**
