# Supplementary material for: Ring distributions leading to species formation: a global topographic analysis of geographic barriers associated with ring species
Source: BMC Biol. 2012 Mar 12;10:20. doi: 10.1186/1741-7007-10-20 (PMC3320551; doi:10.1186/1741-7007-10-20)
Supplement: Additional file 6 — Use of the topographic ring model to identify candidate taxa for ring diversification around a focal barrier in the Iberian Peninsula (southern Europe) that is topographically similar to the reference barrier for the Drakensberg Massif (South Africa), which has promoted ring diversification in a tree species, Acacia karroo. Extensive field-based studies in Iberia - particularly in reptiles and amphibians - have generated considerable distributional and phylogeographic data that can be used to evaluate whether the focal barrier has promoted continuous levels of differentiation typical of ring divergence. The focal barrier (top panel, map) is a long-standing geographic barrier for terrestrial organisms, serving as a steep ecotone between the main climatic regions of Iberia. As a result of its particular topography, a central arid and warmer plateau is surrounded by moister and colder habitat. These climatic conditions have shaped the distribution of many Atlantic species on the peninsula, including the fire salamanders Salmandra salamandra, and also Schreiber's green lizard Lacerta schreiberi, which forms a nearly complete ring distribution around the barrier (map). Extensive genetic data (in both mitochondrial and nuclear DNA) have been collected to reconstruct its phylogeographic history. In agreement with our model prediction, multi-locus data suggest that the focal barrier has strongly influenced non-adaptive divergence among currently contiguous populations of L. schreiberi, showing evidence of continuous levels of genetic differentiation around the barrier and no evidence of historical gene flow across it (bottom panel, phylogenetic network; thick branches are supported by > 0.95 posterior probability). Although the species in this example lacks terminal overlap, it illustrates how the topographic ring model may be used to properly identify and evaluate new instances of ring diversification. [file 1741-7007-10-20-S6.PDF]

Monahan, Pereira, & Wake: Ring Distributions Leading to Species Formation
Additional file 6

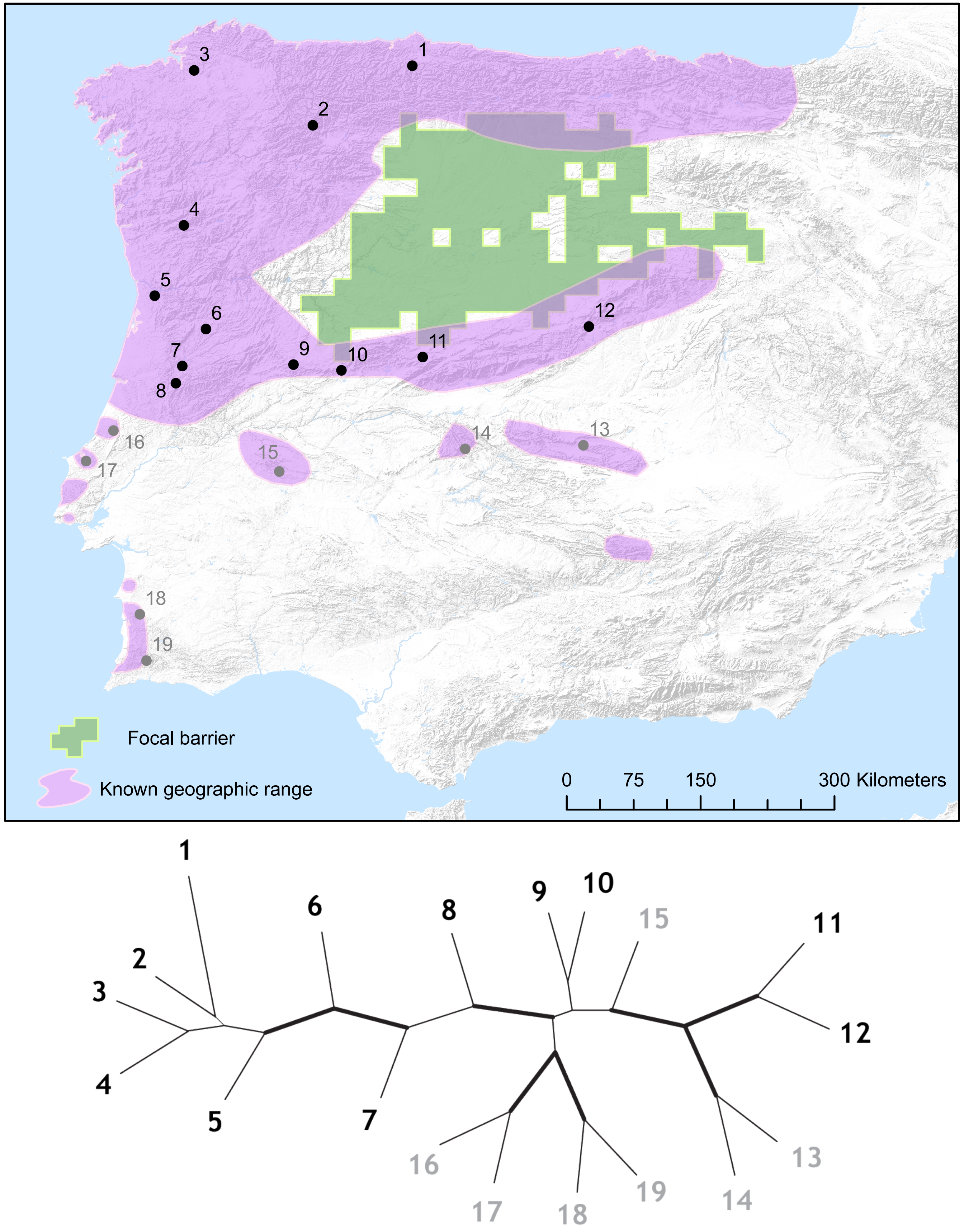

Use of the topographic ring model to identify candidate taxa for ring diversification around a focal barrier in the Iberian Peninsula (southern Europe) that is topographically similar to the reference barrier for the Drakensberg Massif (South Africa), which has promoted ring diversification in a tree species, *Acacia karroo* [1]. Extensive field-based studies in Iberia – particularly in reptiles and amphibians – have generated considerable distributional [2,3] and phylogeographic [4] data that can be used to evaluate whether the focal barrier has promoted continuous levels of differentiation typical of ring divergence. The focal barrier (top panel, map) is a long-standing geographic barrier for terrestrial organisms, serving as a steep ecotone between the main climatic regions of Iberia [5]. As a result of its particular topography, a central arid and warmer plateau is surrounded by moister and colder habitat. These climatic conditions have shaped the distribution of many Atlantic species on the peninsula [5], including the fire salamanders *Salmandra salamandra* [6], and also Schreiber's green lizard *Lacerta schreiberi* [7], which forms a nearly complete ring distribution around the barrier (map). Extensive genetic data (in both mitochondrial and nuclear DNA) have been collected to reconstruct its phylogeographic history [7]. In agreement with our model prediction, multi-locus data suggest that the focal barrier has strongly influenced non-adaptive divergence among currently contiguous populations of *L. schreiberi*, showing evidence of continuous levels of genetic differentiation around the barrier and no evidence of historical gene flow across it (bottom panel, phylogenetic network; thick branches are supported by >0.95 posterior probability). Although the species in this example lacks terminal overlap, it illustrates how the topographic ring model may be used to properly identify and evaluate new instances of ring diversification. Genetic data and sampling locations adapted from Godinho et al. [7]. Geographic range map obtained from IUCN [8].

References

1. Brain P: **Genetic races in a ring species, *Acacia karroo*.** *S Afr J Sci* 1989, **85**:181-185.
2. Pleguezuelos JM, Márquez R, Lizana M: *Atlas de Distribución y Libro Rojo de los Anfibios y Reptiles de España, 2a Impresión*. Madrid: Dirección General de Conservación de la Naturaleza-Asociación Herpetológica Española; 2002.
3. Loureiro A, Ferrand de Almeida N, Carretero M, Paulo OS: *Atlas dos Anfíbios e Répteis de Portugal*. Lisboa: Instituto da Conservação da Natureza e Biodiversidade; 2010.
4. Gómez A, Lunt D: In *Phylogeography in Southern European Refugia*. Edited by Weiss S, Ferrand N. Dordrecht, The Netherlands Springer; 2007:155-188.
5. Sillero N, Brito JC, Skidmore AK, Toxopeus AG: **Biogeographical patterns derived from remote sensing variables: the amphibians and reptiles of the Iberian Peninsula.** *Amphibia-Reptilia* 2009, **30**:185-206.
6. García-París M, Alcobendas M, Buckley D, Wake DB: **Dispersal of viviparity across contact zones in Iberian populations of fire salamanders (*Salamandra*) inferred from discordance of genetic and morphological traits.** *Evolution* 2003, **57**:129-143.
7. Godinho R, Crespo EG, Ferrand N: **The limits of mtDNA phylogeography: complex patterns of population history in a highly structured Iberian lizard are only revealed by the use of nuclear markers.** *Molec Ecol* 2008, **17**:4670-4683.
8. IUCN: **IUCN Red List of Threatened Species, version 2009.1.**
