# Supplementary material for: Ring distributions leading to species formation: a global topographic analysis of geographic barriers associated with ring species
Source: BMC Biol. 2012 Mar 12;10:20. doi: 10.1186/1741-7007-10-20 (PMC3320551; doi:10.1186/1741-7007-10-20)
Supplement: Additional file 7 — Use of the topographic ring model to identify candidate taxa for ring diversification around a focal barrier near the Baja California Peninsula (USA and Mexico) that is topographically similar to the reference barrier for the Drakensberg Massif (South Africa), which has promoted ring diversification in a tree species, Acacia karroo. The focal barrier (left panel, map) is a low-lying topographic depression located at the land-sea interface in the northern Sea of Cortez. As a result of its particular topography, the barrier has promoted diversification in a number of terrestrial taxa, including Hypsiglena nightsnakes and the rosy boa Lichanura trivirgata. In L. trivirgata, mitochondrial data have been collected to reconstruct its phylogeographic history. In agreement with our model prediction, these data suggest that the focal barrier has strongly influenced non-adaptive divergence among mostly contiguous subspecies of L. trivirgata, showing evidence of continuous levels of genetic differentiation along either side of the barrier (right panel, phylogenetic network; thick branches are supported by > 0.95 posterior probability). Closure of the ring distribution may occur in the northwest, between two deeply divergent lineages within the subspecies roseofusca (symbolized by circles and hexagons). [file 1741-7007-10-20-S7.PDF]

Monahan, Pereira, & Wake: Ring Distributions Leading to Species Formation
Additional file 7

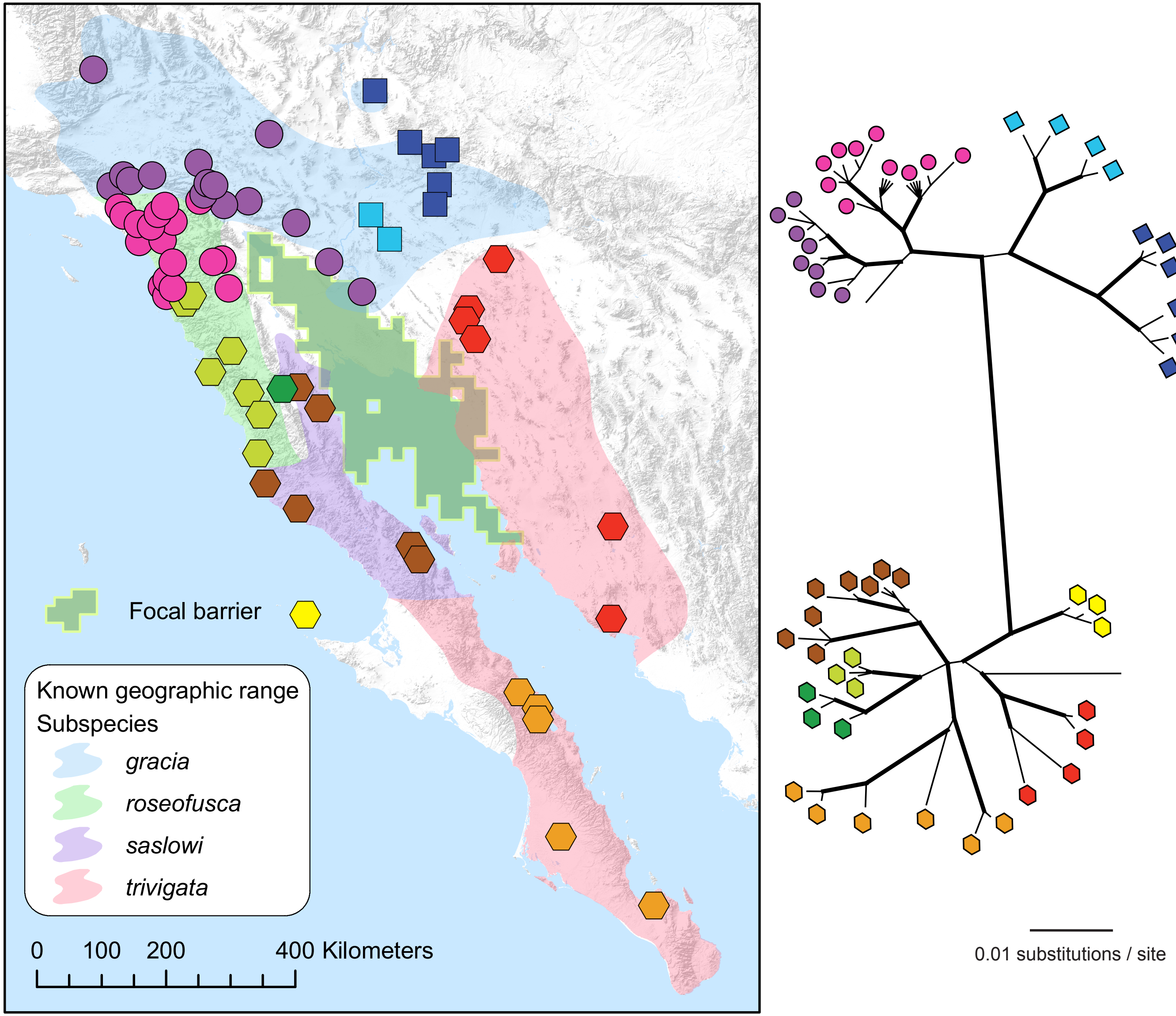

Use of the topographic ring model to identify candidate taxa for ring diversification around a focal barrier near the Baja California Peninsula (USA and Mexico) that is topographically similar to the reference barrier for the Drakensberg Massif (South Africa), which has promoted ring diversification in a tree species, *Acacia karroo* [1]. The focal barrier (left panel, map) is a low-lying topographic depression located at the land-sea interface in the northern Sea of Cortez. As a result of its particular topography, the barrier has promoted diversification in a number of terrestrial taxa, including *Hypsiglena* nightsnakes [2] and the rosy boa *Lichanura trivirgata* [3,4]. In *L. trivirgata*, mitochondrial data have been collected to reconstruct its phylogeographic history [4]. In agreement with our model prediction, these data suggest that the focal barrier has strongly influenced non-adaptive divergence among mostly contiguous subspecies of *L. trivirgata*, showing evidence of continuous levels of genetic differentiation along either side of the barrier (right panel, phylogenetic network; thick branches are supported by >0.95 posterior probability). Closure of the ring distribution may occur in the northwest, between two deeply divergent lineages within the subspecies *roseofusca* (symbolized by circles and hexagons). Genetic data and sampling locations adapted from Wood et al. [4]. Geographic range map obtained from IUCN [5], with subspecies distributions added based on Stebbins [3] and Wood et al. [4].

References

1. Brain P: **Genetic races in a ring species, *Acacia karroo*.** *S Afr J Sci* 1989, **85**: 181-185.
2. Mulcahy D, Macey J: **Vicariance and dispersal form a ring distribution in nightsnakes around the Gulf of California.** *Mol Phylogenet Evol* 2009: **53**:537-546.
3. Stebbins RC: *A Field Guide to Western Reptiles and Amphibians*. New York: Houghton Mifflin; 2003.
4. Wood DA, Fisher RN, Reeder TW: **Novel patterns of historical isolation, dispersal, and secondary contact across Baja California in the Rosy Boa (*Lichanura trivirgata*).** *Mol Phylogenet Evol* 2008: **46**:484-502.
5. IUCN: **IUCN Red List of Threatened Species, version 2009.1.**

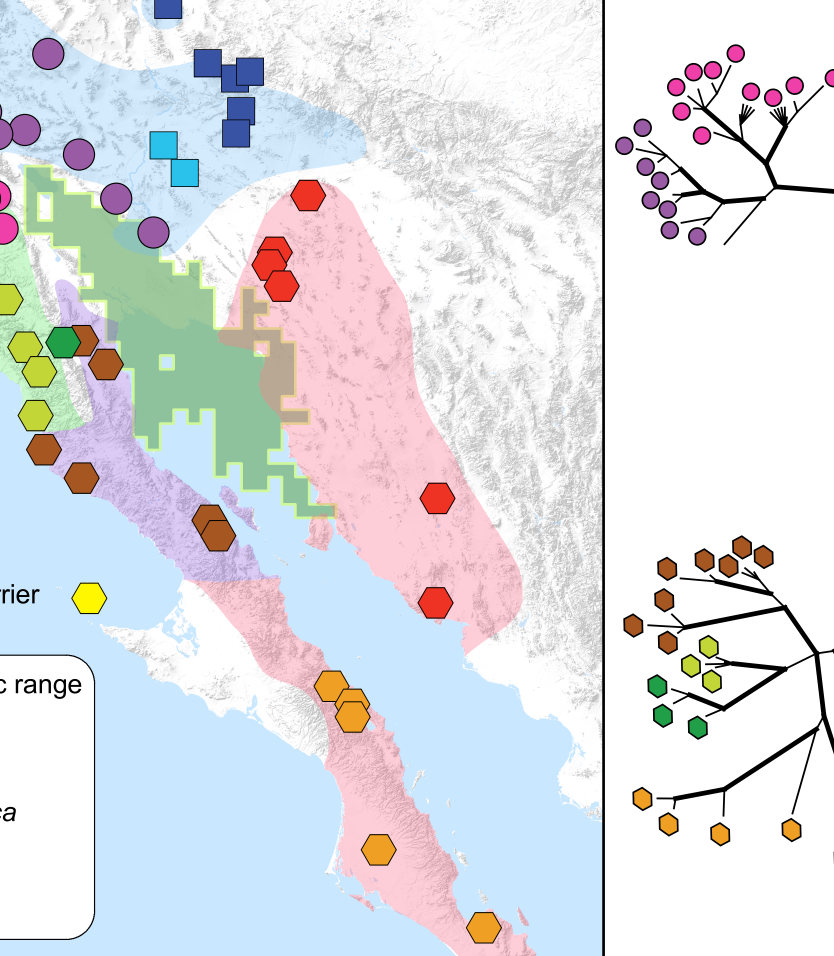
rrier
c range
ca

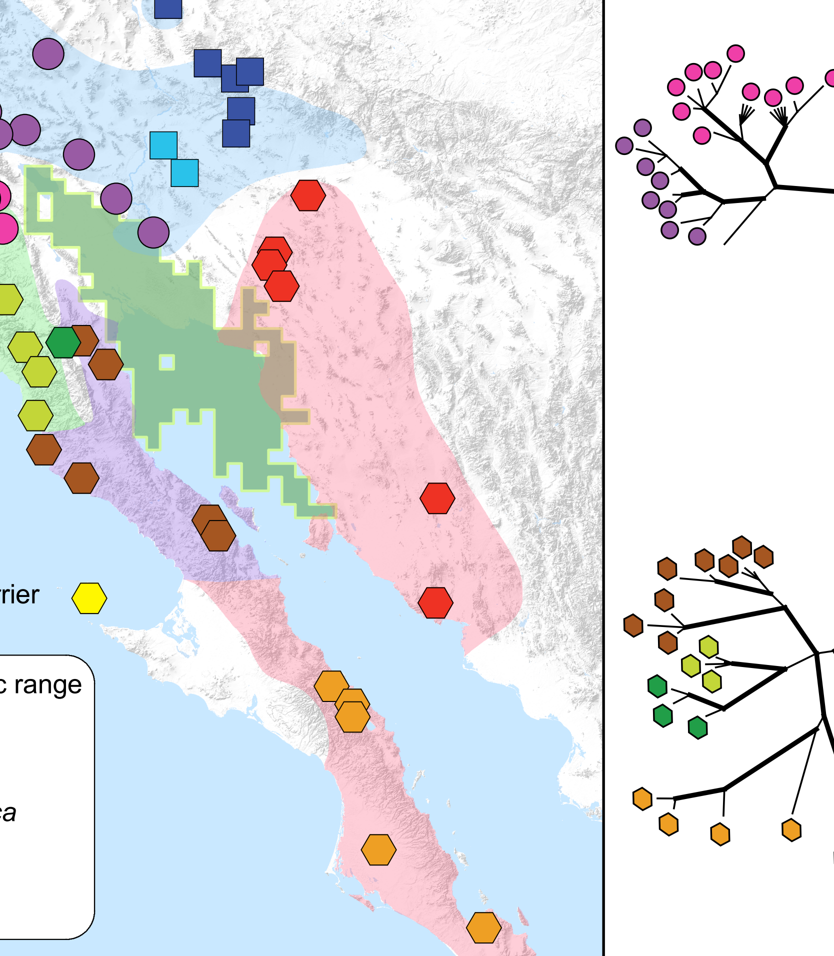
rrier
c range
ca
