# Supplementary material for: Ring distributions leading to species formation: a global topographic analysis of geographic barriers associated with ring species
Source: BMC Biol. 2012 Mar 12;10:20. doi: 10.1186/1741-7007-10-20 (PMC3320551; doi:10.1186/1741-7007-10-20)
Supplement: Additional file 8 — Use of the topographic ring model to identify candidate taxa for ring diversification around a focal barrier on the island of New Guinea that is topographically similar to the reference barrier for the Central Valley (California, USA), which has promoted ring diversification in a salamander, Ensatina eschscholtzii. The focal barrier (upper right panel, map) is a mountain forest ecoregion that is surrounded at lower elevations by warmer and generally drier ecoregions and basins. This distribution of contrasting bioclimates is hypothesized to have promoted diversification in a number of bird taxa, including Pitohui, Tanysiptera kingfishers, Aegotheles owlet-nightjars, and the little shrike-thrush Colluricincla megarhyncha. All of these taxa are monophyletic and have diversified around the barrier, reaching different stages of divergence. This diversification is especially well illustrated by C. megarhyncha, where mitochondrial data have been collected to reconstruct its phylogeographic history. In agreement with our model prediction, these data suggest that the focal barrier has strongly influenced non-adaptive divergence among mostly contiguous subspecies of C. megarhyncha, showing evidence of continuous levels of genetic differentiation along either side of the barrier (left panel, phylogenetic network; numbers report numbers of site or base pair substitutions between haplotypes). Additionally, plots of genetic vs. geographic distance (lower right panel, plot) reveal significant isolation by distance around the barrier, but not across it, suggesting that this is an important barrier to colonization and gene flow. Although it is unclear whether there is terminal overlap at the southern end of the ring distribution, this example illustrates how the topographic ring model may be used to properly identify and evaluate new instances of ring diversification. [file 1741-7007-10-20-S8.PDF]

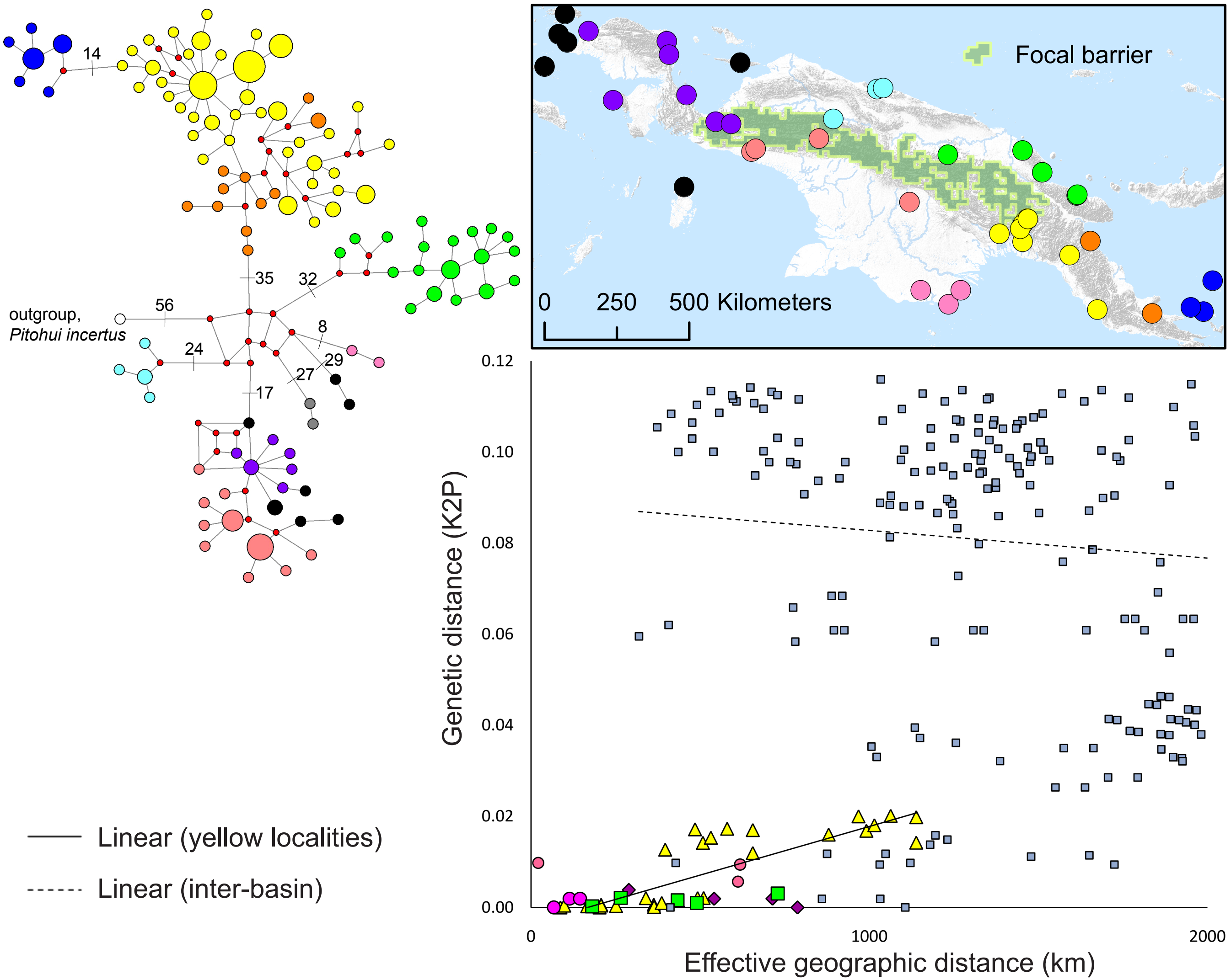

Use of the topographic ring model to identify candidate taxa for ring diversification around a focal barrier on the island of New Guinea that is topographically similar to the reference barrier for the Central Valley (California, USA), which has promoted ring diversification in a salamander, *Ensatina eschscholtzii* [1]. The focal barrier (upper right panel, map) is a mountain forest ecoregion that is surrounded at lower elevations by warmer and generally drier ecoregions and basins. This distribution of contrasting bioclimates is hypothesized to have promoted diversification in a number of bird taxa, including *Pitohui* [2], *Tanysiptera* kingfishers [3,4], *Aegotheles* owlet-nightjars [5], and the little shrike-thrush *Colluricincla megarhyncha* [6]. All of these taxa are monophyletic and have diversified around the barrier, reaching different stages of divergence. This diversification is especially well illustrated by *C. megarhyncha*, where mitochondrial data have been collected to reconstruct its phylogeographic history [6]. In agreement with our model prediction, these data suggest that the focal barrier has strongly influenced non-adaptive divergence among mostly contiguous subspecies of *C. megarhyncha*, showing evidence of continuous levels of genetic differentiation along either side of the barrier (left panel, phylogenetic network; numbers report numbers of site or base pair substitutions between haplotypes). Additionally, plots of genetic vs. geographic distance (lower right panel, plot) reveal significant isolation by distance around the barrier, but not across it, suggesting that this is an important barrier to colonization and gene flow. Although it is unclear whether there is terminal overlap at the southern end of the ring distribution, this example illustrates how the topographic ring model may be used to properly identify and evaluate new instances of ring diversification. Genetic data and sampling locations adapted from Deiner et al. [6].

References

1. Wake DB: **Incipient species formation in salamanders of the *Ensatina complex*.** *Proc Natl Acad Sci USA* 1997, **94**:7761-7767.
2. Dumbacher JP, Fleischer RC: **Phylogenetic evidence for colour pattern convergence in toxic pitohuis: Müllerian mimicry in birds?** *Proc R Soc Lond B* 2001, **268**:1971-1976.
3. Mayr E: *Systematics and the Origin of Species, from the Viewpoint of a Zoologist*. Cambridge, MA: Harvard University Press; 1942.
4. Mayr E: *Animal Species and Evolution*. Cambridge, MA: Belknap Press; 1963.
5. Dumbacher JP, Pratt TK, Fleischer RC: **Phylogeny of the owlet-nightjars (Aves: Aegothelidae) based on mitochondrial DNA sequence.** *Mol Phylogenet Evol* 2003, **29**:540-549.
6. Deiner K, Lemmon AR, Mack AL, Fleischer RC, Dumbacher JP: **A Passerine bird's evolution corroborates the geologic history of the island of New Guinea.** *PLoS ONE* 2011, **6**:e19479.
