# Supplementary material for: Ring distributions leading to species formation: a global topographic analysis of geographic barriers associated with ring species
Source: BMC Biol. 2012 Mar 12;10:20. doi: 10.1186/1741-7007-10-20 (PMC3320551; doi:10.1186/1741-7007-10-20)
Supplement: Additional file 9 — Use of the topographic ring model to identify candidate taxa for ring diversification around a focal barrier in Madagascar that is topographically "in between" (Figure 3) reference barriers for the Drakensberg Massif (South Africa), which has promoted ring diversification in a tree species, Acacia karroo, and the Tibetan Plateau (Central Asia), which has promoted ring diversification in a bird species, Phylloscopus trochiloides. The focal barrier (right panel, map) is a mountainous subhumid bioclimatic zone surrounded at lower elevations by humid (east) and subarid/dry (west) zones. This distribution of contrasting bioclimates is hypothesized to have promoted diversification in amphibians, reptiles, and lemurs, some of which form either complete or nearly complete ring distributions around the barrier. In Propithecus lemurs, mitochondrial data have been collected to reconstruct its phylogeographic history. In agreement with our model prediction, these data suggest that the focal barrier has strongly influenced non-adaptive divergence among mostly contiguous species of Propithecus, showing evidence of continuous levels of genetic differentiation (from north to south) along either side of the barrier (left panel, phylogenetic tree; thick branches are supported by > 0.95 posterior probability). Although there appears to be no overlap of terminal taxa in the south, this example illustrates how the topographic ring model may be used to properly identify and evaluate new instances of ring diversification. [file 1741-7007-10-20-S9.PDF]

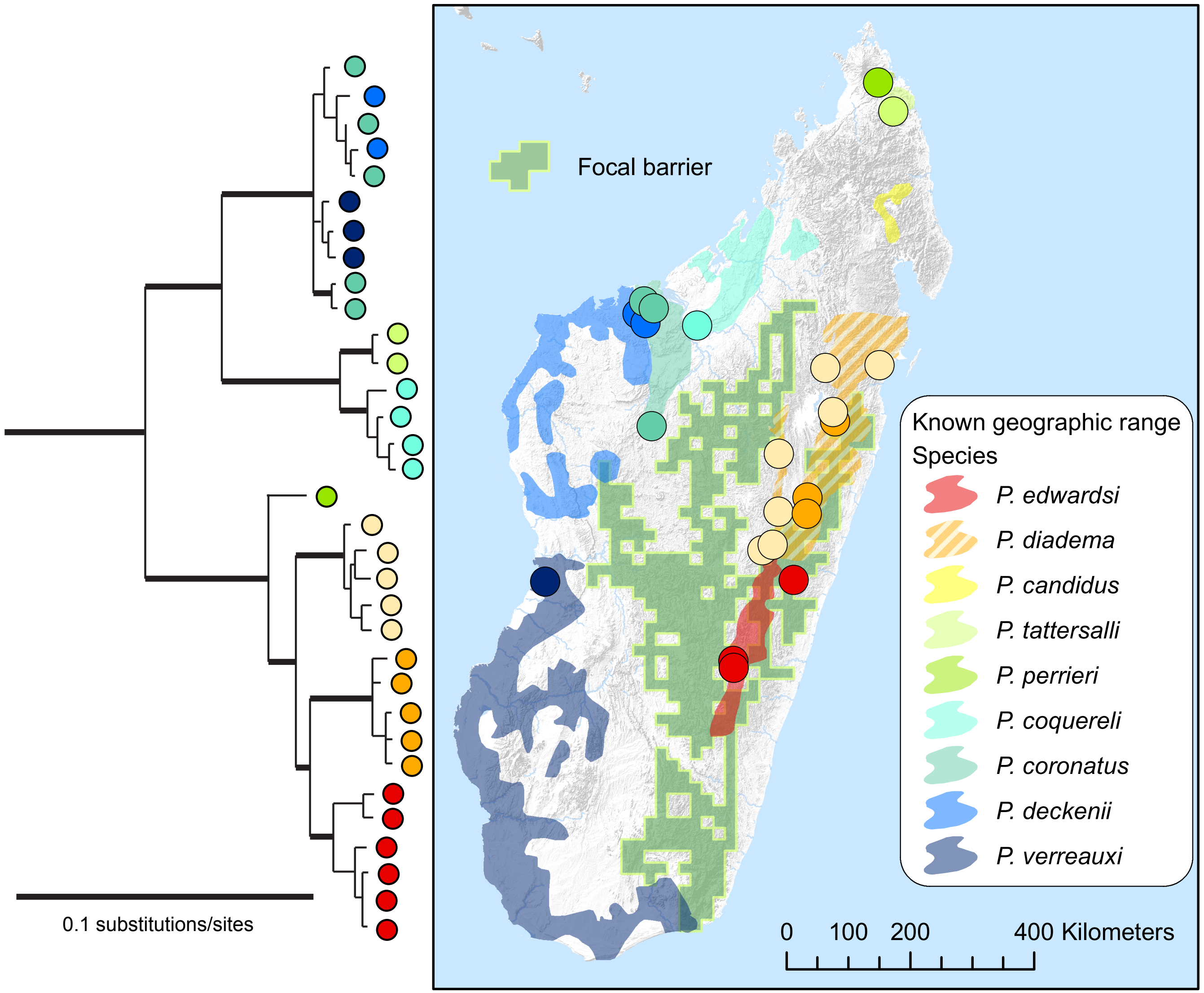

Use of the topographic ring model to identify candidate taxa for ring diversification around a focal barrier in Madagascar that is topographically "in between" (Figure 3) reference barriers for the Drakensberg Massif (South Africa), which has promoted ring diversification in a tree species, *Acacia karroo* [1], and the Tibetan Plateau (Central Asia), which has promoted ring diversification in a bird species, *Phylloscopus trochiloides* [2]. The focal barrier (right panel, map) is a mountainous subhumid bioclimatic zone surrounded at lower elevations by humid (east) and subarid/dry (west) zones [3]. This distribution of contrasting bioclimates is hypothesized to have promoted diversification in amphibians [4], reptiles [5], and lemurs [6-8], some of which form either complete or nearly complete ring distributions around the barrier. In *Propithecus* lemurs, mitochondrial data have been collected to reconstruct its phylogeographic history [8]. In agreement with our model prediction, these data suggest that the focal barrier has strongly influenced non-adaptive divergence among mostly contiguous species of *Propithecus*, showing evidence of continuous levels of genetic differentiation (from north to south) along either side of the barrier (left panel, phylogenetic tree; thick branches are supported by >0.95 posterior probability). Although there appears to be no overlap of terminal taxa in the south, this example illustrates how the topographic ring model may be used to properly identify and evaluate new instances of ring diversification. Genetic data and sampling locations adapted from Rumpler et al. [8]. Geographic range map obtained from IUCN [9].

References

1. Brain P: **Genetic races in a ring species, *Acacia karroo*.** *S Afr J Sci* 1989, **85**:181-185.
2. Irwin DE, Bensch S, Price TD: **Speciation in a ring.** *Nature* 2001, **409**:333-337.
3. Vences M, Wollenberg KC, Vieites DR, Lees DC: **Madagascar as a model region of species diversification.** *Trends Ecol Evol* 2009, **24**:456-465.
4. Vieites DR, Wollenberg KC, Andreone F, Köhler J, Glawe F, Vences M: **Vast underestimation of Madagascar's biodiversity evidenced by an integrative amphibian inventory.** *Proc Natl Acad Sci USA* 2009, **106**:8267-8272.
5. Boumans L, Vieites DR, Glaw F, Vences M: **Geographical patterns of deep mitochondrial differentiation in widespread Malagasy reptiles.** *Mol Phylogenet Evol* 2007, **45**:822-839.
6. Yoder AD, Heckman KL: In *Primate Biogeography: Progress and Prospects*. Edited by Lehman SM, Fleagle JG. New York: Springer Science; 2006:255-268.
7. Weisrock DW, Rasoloarison RM, Fiorentino I, Ralison JM, Goodman SM, Kappeler PM, Yoder AD: **Delimiting species without nuclear monophyly in Madagascar's mouse lemurs.** *PLoS ONE* 2010, **5**:e9883.
8. Rumpler Y, Hauwy M, Fausser J-L, Roos C, Zaramody A, Andriaholinirina N, Zinner D: **Comparing chromosomal and mitochondrial phylogenies of the Indriidae (Primates, Lemuriformes).** *Chromosome Res* 2011, **19**:209-24.
9. IUCN: **IUCN Red List of Threatened Species, version 2009.1.**
